# mTORC1 promotes malignant large cell/anaplastic histology and is a targetable vulnerability in SHH-*TP53* mutant medulloblastoma

**DOI:** 10.1172/jci.insight.153462

**Published:** 2021-12-08

**Authors:** Valentina Conti, Manuela Cominelli, Valentina Pieri, Alberto L. Gallotti, Ilaria Pagano, Matteo Zanella, Stefania Mazzoleni, Flavia Pivetta, Monica Patanè, Giulia M. Scotti, Ignazio S. Piras, Bianca Pollo, Andrea Falini, Alessio Zippo, Antonella Castellano, Roberta Maestro, Pietro L. Poliani, Rossella Galli

**Affiliations:** 1 Neural Stem Cell Biology Unit, Division of Neuroscience, San Raffaele Scientific Institute, Milan, Italy.; 2 Pathology Unit, Molecular and Translational Medicine Department, University of Brescia, Brescia, Italy.; 3 Functional Neuroradiology Unit, Vita-Salute San Raffaele University and San Raffaele Scientific Institute, Milan, Italy.; 4 Istituto Nazionale di Genetica Molecolare (INGM), Milan, Italy.; 5 Unit of Experimental Oncology 1, Centro di Riferimento Oncologico (CRO), Aviano National Cancer Institute, Aviano, Pordenone, Italy.; 6 Neuropathology Unit, Fondazione IRCCS Istituto Neurologico “C. Besta,” Milan, Italy.; 7 Center for Omics Sciences, San Raffaele Scientific Institute, Milan, Italy.; 8 Neurogenomics Division, Translational Genomics Research Institute (TGen), Phoenix, Arizona, USA.; 9 Laboratory of Chromatin Biology & Epigenetics, Department of Cellular, Computational and Integrative Biology (CIBIO), University of Trento, Trento, Italy.

**Keywords:** Neuroscience, Oncology, Brain cancer, Mouse models

## Abstract

Medulloblastoma (MB), one of the most malignant brain tumors of childhood, comprises distinct molecular subgroups, with p53 mutant sonic hedgehog–activated (SHH-activated) MB patients having a very severe outcome that is associated with unfavorable histological large cell/anaplastic (LC/A) features. To identify the molecular underpinnings of this phenotype, we analyzed a large cohort of MB developing in p53-deficient *Ptch^+/–^* SHH mice that, unexpectedly, showed LC/A traits that correlated with mTORC1 hyperactivation. Mechanistically, mTORC1 hyperactivation was mediated by a decrease in the p53-dependent expression of mTORC1 negative regulator Tsc2. Ectopic mTORC1 activation in mouse MB cancer stem cells (CSCs) promoted the in vivo acquisition of LC/A features and increased malignancy; accordingly, mTORC1 inhibition in p53-mutant *Ptch^+/–^* SHH MB and CSC-derived MB resulted in reduced tumor burden and aggressiveness. Most remarkably, mTORC1 hyperactivation was detected only in p53-mutant SHH MB patient samples, and treatment with rapamycin of a human preclinical model phenocopying this subgroup decreased tumor growth and malignancy. Thus, mTORC1 may act as a specific druggable target for this subset of SHH MB, resulting in the implementation of a stringent risk stratification and in the potentially rapid translation of this precision medicine approach into the clinical setting.

## Graphical abstract

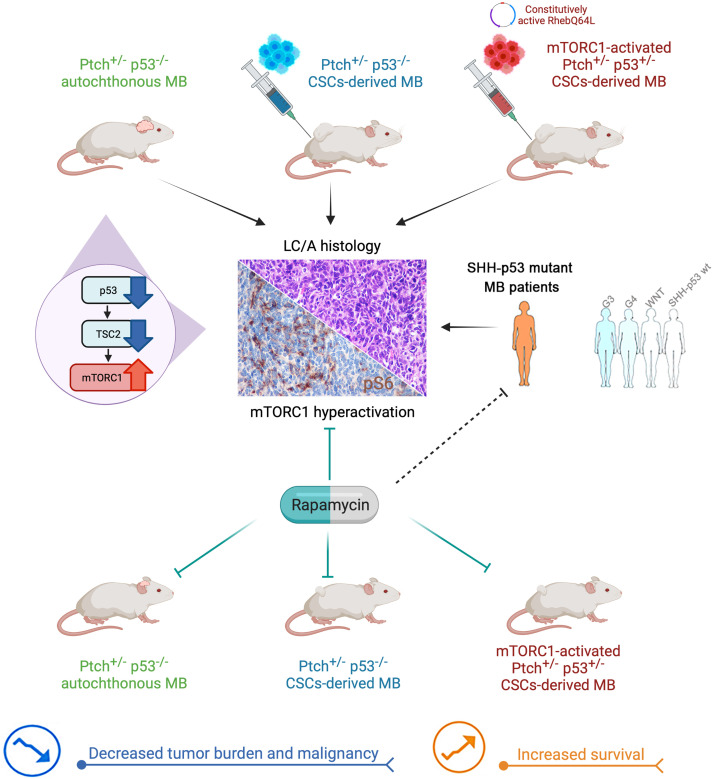

## Introduction

Medulloblastoma (MB) is one of the most common malignant brain tumors of children, thought to originate from distinct neural stem/progenitor cell populations of the cerebellum during early embryonic development (1, 2). The peak age of diagnosis is between 6 and 8 years of age, although MB can also occur during the first years of life or during adulthood (1).

Genetic profiling identified different molecular consensus subgroups of MB (i.e., wingless and Int1–activated [WNT-activated], sonic hedgehog–activated [SHH-activated], Group 3, and Group 4) (3), which were further subclassified into 7–12 molecular subtypes (4, 5). Importantly, key molecular alterations identified in MB subgroups have inferred the development of potentially novel biomarkers that were confirmed in the fifth edition of the World Health Organization (WHO) Classification of Tumors of the Central Nervous System (WHO CNS5), which classifies MB not only based on their histological appearance, but also on their molecular features, and identifies 4 molecular variants of the disease (WNT, SHH-*TP53* WT, SHH–*TP53* mutant (SHH-*TP53*mut, and non-WNT/non-SHH, the latter including both Group 3 and Group 4 MB; refs. 6, 7).

Generally, WNT subgroup patients have an excellent prognosis, whereas Group 3 patients have a substantially worse prognosis (3, 8, 9). Most remarkably, SHH subgroup patients, who are associated with an intermediate prognosis, have a profoundly worse outcome when presenting with mutations in p53, due to the association with catastrophic cellular events (i.e., chromothrypsis) and cellular anaplasia (6, 10, 11).

The current standard-of-care treatment for MB is known to lead to severe neurocognitive and neuroendocrine sequelae. Although SHH pathway inhibitors have shown promise in SHH MB in early-phase clinical trials, treatment of infants and young children with these inhibitors needs to be approached with caution because of the risk of skeletal defects (6). In addition, SHH inhibitors such as Smoothened antagonists are subjected to resistance development (12, 13). Thus, the development of lowly toxic and, possibly, subgroup/subtype-tailored targeted therapeutic approaches is strongly needed.

Among the different SHH MB molecular subtypes, very high–risk patients, such as those in the SHH subset of patients with mutated *TP53*, large cell/anaplastic (LC/A) histology and metastatic disease (also described as SHH_children_ or SHHα subtypes; refs. 4, 5), are typically refractory to both conventional and SHH-targeted therapies and, therefore, should be prioritized for alternative upfront treatment strategies (6, 11).

Although the majority of SHH MB with mutant *TP53* show amplification of *MYCN* and/or *GLI2* (14), there are reports of rare SHH-*TP53*mut MB, which are classified as LC/A SHHα and are characterized by concurrent mutations in *PTCH1* and *TP53* (11, 15). Notwithstanding these findings, several preclinical models of MB that do not bear the same exact genetic mutations found in humans are still reminiscent of many critical and relevant molecular and phenotypic features of human MB (16–18).

With this in mind, we subjected autochthonous SHH MB developing in *Ptch1*^+/–^ *p53*^–/–^ (HN) mice (19) to a comprehensive and detailed histological and molecular analysis to delineate whether features of high-risk LC/A SHH-*TP53*mut MB could be detected in these mice, thus making them potential phenotypic proxies of this MB variant.

Notably, we identified a previously undetected large subset of *Ptch1*^+/–^ *p53*^–/–^ mice showing LC/A morphological features that we prove to significantly correlate with aberrant activation of the mTOR signaling pathway. This correlation was found not only in preclinical models, but also — and most relevantly — in human SHH-*TP53*mut MB specimens from a collection of 90 human MB samples affiliated with all 4 molecular subgroups.

The activation and role of the mTOR pathway has been previously investigated in MB, with many studies reporting its potential targetability in vitro and in vivo by means of different mouse and human preclinical approaches (20–24). However, the significance of mTORC1 hyperactivation in specific molecular subtypes, such as the SHH *TP53*mut subtype of MB, has been poorly explored.

By taking advantage of subgroup-specific SHH, WNT, and Group 3 mouse cancer stem cell (CSC) lines that others and we isolated by the NeuroSphere assay from different subtypes of mouse MB (17, 18, 25, 26), we report for the first time to our knowledge that mTOR hyperactivation in MB is causally and specifically involved in the acquisition of LC/A histology and in increased malignancy in a specific subset of human SHH MB; we also note that addiction to this pathway in these patients may represent a cancer-specific vulnerability to be taken advantage of therapeutically.

## Results

### Autochthonous MB developing in Ptch1^+/–^ p53^–/–^ mice show LC/A histopathological features that are associated with mTORC1 hyperactivation.

*Ptch1*^+/–^*p53*^+/+^ (abbreviated to heterozygous/WT [HW]) mice develop MB with 30%–40% frequency over an average period of 5 months (Supplemental Figure 1; supplemental material available online with this article; https://doi.org/10.1172/jci.insight.153462DS1). Conversely, *Ptch1*^+/–^ *p53*^–/–^ (heterozygous/null [HN]) mice generate MB with full penetrance and shorter time to tumor formation; in fact, they succumb to MB within 2 months after birth (Supplemental Figure 1).

**Figure 1:**
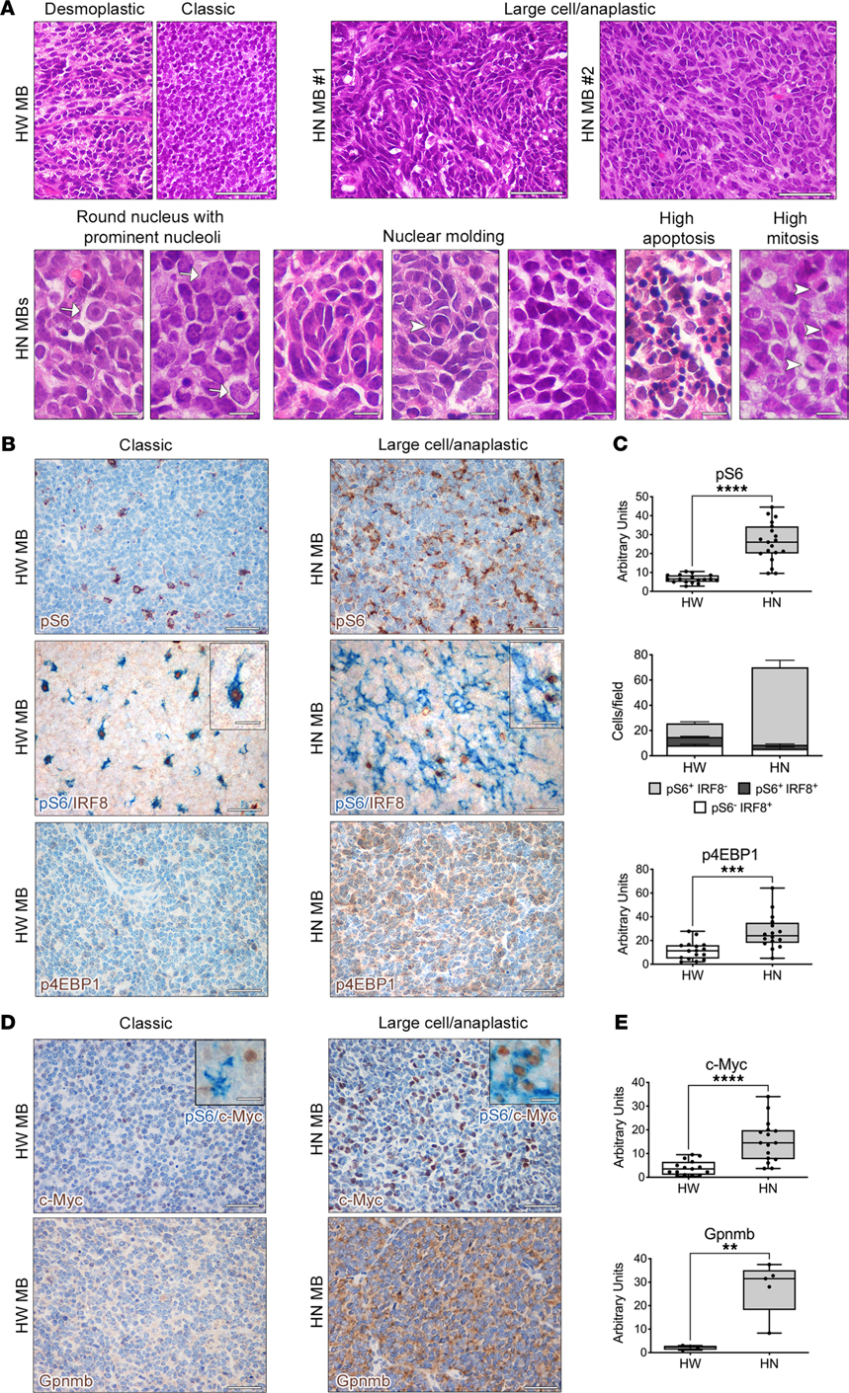
Autochthonous MB developing in *Ptch1*^+/–^ *p53*^–/–^ mice show large cell/anaplastic histopathological features that are associated with mTORC1 hyperactivation. (**A**) Typical features of desmoplastic MB (e.g., small undifferentiated cells interspersed in a dense neuropil) and of classic MB (e.g., small “blue” cells with round/ovoid nuclei) are found in HW MB (H&E). Conversely, histomorphological LC/A characteristics are observed in HN MB. Scale bars: 50 μm. Traits typical of the large cell variant, as cells with round nuclei with prominent nuclei (white arrows), and of the anaplastic variant, as angular nuclei, nuclear wrapping (white arrowhead), nuclear molding, and frequent apoptotic and mitotic cells (white arrowheads), are detected in all HN MB. Scale bars: 10 μm. (**B**) Higher numbers of cells hyperactivating pS6 (cytoplasmic staining, brown) are found in HN LC/A MB than in HW MB. The few pS6-IR cells (cytoplasmic staining, blue) in HW MB are stromal cells, positive for the macrophage/microglia marker IRF8 (nuclear staining, brown). Conversely, most pS6-IR cells in HN MB (cytoplasmic staining, blue) are tumor cells that do not express IRF8 (nuclear staining, brown). Increased activation of p4EBP1 is observed in HN MB (cytoplasmic staining, brown). Scale bars: 50 μm; insets: 10 μm. (**C**) Quantification of marker expression (shown as arbitrary units) and of cell subpopulations (shown as number of cells in a 60× microscopic field) is shown in the graphs. pS6^+^IRF8^–^ cells in HW versus HN MB: ***P < 0.005, ****P < 0.0001, Student’s *t* test, unpaired. (**D**) Increased c-Myc expression (nuclear staining, brown) is detected in HN MB, with some c-Myc^+^ cells being also pS6-IR (cytoplasmic, blue; insets). mTORC1-regulated gene Gpnmb (cytoplasmic staining, brown) is upregulated in HN MB. Scale bars: 50 μm; insets: 10 μm. (**E**) Quantification of marker expression is shown in the graphs. **P < 0.01, ****P < 0.0001. Quantitative data are represented as a box-and-whisker plot, with bounds from 25th to 75th percentile, median line, and whiskers ranging from minimum to maximum values. Student’s *t* test, unpaired. **P < 0.01; ***P < 0.005; ****P < 0.001.

To date, a thorough histomorphological classification of these different MB has not been performed. To this end, we subjected a large collection of MB specimens, obtained from littermates of the 2 genotypes, to histological analysis (Supplemental Table 1, A and B). H&E staining indicated that 91% of the HW MB analyzed (*n* = 52 of 57) showed desmoplastic traits, such as the presence of small undifferentiated cells with hyperchromatic nuclei, maturation of cells with abundant cytoplasm and dense intercellular neuropil, or the appearance of classic features, such as the presence of densely packed, small “blue” round cells (Figure 1A).

Unexpectedly, all MB developing in HN mice were endowed with the characteristics of LC/A histology, which corresponds to the most malignant histological variant of human MB (ref. 27 and Supplemental Table 1). In fact, 100% (*n* = 21 of 21) of HN mice harbored tumors with high numbers of cells displaying marked nuclear pleomorphism (Figure 1A). Different types of large cells were observed — e.g., discohesive cells with round nuclei and prominent nucleoli (large cell subtype) or highly atypical nuclei with irregular profile (anaplastic subtype) — resulting in typical features such as nuclear wrapping and nuclear molding (Figure 1A). Apoptotic cells and several mitotic figures were identified in all tumors with LC/A histology (Figure 1A). All these features are reminiscent of those characterizing the majority of human SHH-*TP53*mut MB (10).

To investigate the mechanisms underlying the acquisition of these histological phenotypes, we assessed the activation of a signaling pathway that regulates cell size — i.e., mTORC1 — by testing the phosphorylated form of its surrogate marker ribosomal protein S6_S235/236_ (pS6_S235/236_) in samples from all genotypes by IHC (Figure 1B and Supplemental Table 1, A and B). Tumor cells in HW MB with desmoplastic/classic histology were characterized by low to absent mTORC1 activation (Figure 1, B and C). Indeed, the few pS6-immunoreactive (pS6-IR) cells found in HW MB were mostly tumor-associated inflammatory cells, as shown by positive double staining with the microglia/macrophage nuclear marker IRF8 (ref. 28 and Figure 1, B and C). Conversely, 90% of LC/A HN MB (*n* = 19 of 21) showed many clusters of pS6-IR cells, which were negative for IRF8 expression (Figure 1, B and C), indicating that mTORC1 was hyperactivated in tumor cells only in LC/A MB and might play a role in the acquisition of LC/A features.

To confirm global mTORC1 hyperactivation in HN MB, we also assessed the expression of the other known mTORC1 downstream target, p4EBP1. In line with pS6 activation, also p4EBP1 activation was significantly higher in HN than in HW MB (Figure 1, B and C, and Supplemental Table 1, A and B).

The expression of c-Myc, which has been linked with LC/A phenotypes in Group 3 MB and is also known to be activated by mTORC1, was increased in many cells in LC/A HN MB, with some cells showing concomitant pS6 activation (Figure 1, D and E, and Supplemental Figure 2A).

We also tested the activation of the mTOR complex 2 (mTORC2) pathway and found out that its downstream surrogate marker pNDRG1_T346_ was expressed focally only in a fraction of HN tumors, while being undetectable in HW MB (Supplemental Figure 2B). Likewise, the expression of the known mTORC1-regulated protein glycoprotein nonmetastatic melanoma protein B (Gpnmb) was significantly increased in LC/A HN MB as compared with HW MB (Figure 1, D and E).

### The transcriptional signature of HN MB is reminiscent of that of human SHH MB with p53 mutation.

**Figure 2:**
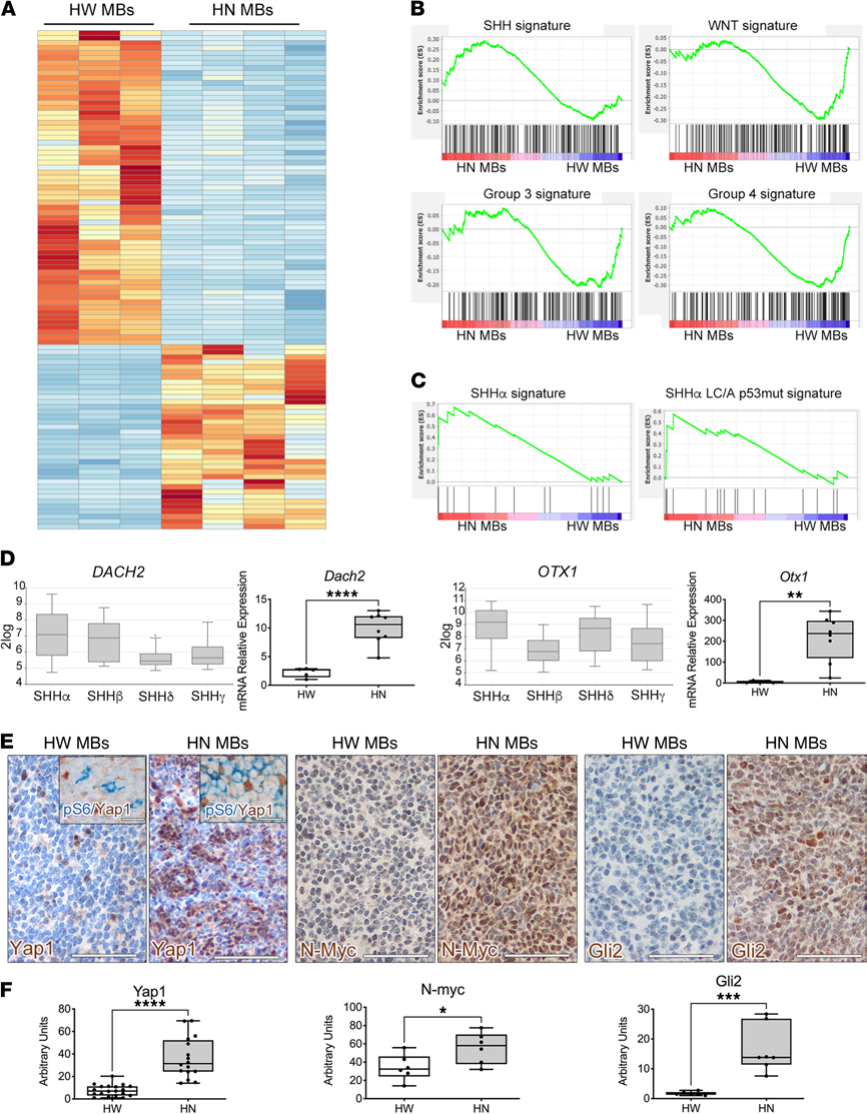
The transcriptional signature of *HN* MB is reminiscent of that of human SHH MB with p53 mutation. (**A**) Supervised whole-transcript expression analysis of HW and HN MB indicates that they are transcriptionally different. (**B**) GSEA indicates that the gene set qualifying the human SHH subgroup is upregulated in HN MB, whereas the gene sets qualifying the human WNT, Group 3, and Group 4 subgroups are upregulated in HW MB. (**C**) The gene sets qualifying the whole SHHα subtype and the SHHα subtype with p53 mutation and LC/A histology are significantly enriched in HN MB. (**D**) *Dach2* and *Otx1* genes, which are significantly upregulated in the human SHHα subtype by in silico analysis of the data set Tumor Medulloblastoma-Cavalli-763 by the R2 software (Genomics Analysis and Visualization Platform, http://r2.amc.nl; *DACH2*: SHHα versus SHHδ: 1.08 × 10^–09^; SHHα versus SHHγ: 1.27 × 10^–07^; *OTX1:* SHHα versus SHHβ: 2.22 × 10^–11^; SHHα versus SHHγ: 5.34 × 10^–06^; Welch *t* test), are highly expressed in HN MB (qPCR). (**E**) The protein abundance of Yap1, N-Myc, and Gli2 (nuclear staining, brown) is significantly enhanced in HN MB, with some Yap1^+^ cells being also immunoreactive for pS6 (cytoplasmic staining, blue). Scale bars: 50 μm; insets: 10 μm. (**F**) Quantification of the level of marker expression is shown in the graphs. Quantitative data are represented as a box-and-whisker plot, with bounds from 25th to 75th percentile, median line, and whiskers ranging from minimum to maximum values. Student’s *t* test, unpaired. *P < 0.05; **P < 0.01; ***P < 0.005; ****P < 0.001.

When compared with HW MB showing desmoplastic/classic histology, LC/A HN tumors were characterized by a distinctive transcriptional signature (Figure 2A and Supplemental List 1, A and B). The differentially expressed genes (DEGs) between desmoplastic/classic HW and LC/A HN MBs were subjected to Gene Set Enrichment Analysis (GSEA) with distinct human transcriptional data sets including the 4 SHH, WNT, Group 3, and Group 4 subgroups, as well as the 4 SHH subtypes (i.e., SHHα, SHHβ, SHHγ, and SHHδ) (5). The gene set qualifying the SHH subgroup of patients versus the other 3 subgroups (WNT, Group 3, and Group 4) was upregulated in LC/A HN tumors (Figure 2B and Supplemental Table 2). Likewise, genes upregulated in the SHHα subtype and, within this subtype, those upregulated in SHHα tumors with p53 mutation and LC/A pathology versus SHHα tumors with WT p53 and desmoplastic/classic histology (5) were again enriched in LC/A HN tumors (Figure 2C, Supplemental Tables 3 and 4, and Supplemental Lists 2 and 3). As an example, *Dach2* and *Otx1* — which were 2 of the 4 genes found in the core enrichment list after performing GSEA on the SHHα gene signature derived from the differential gene expression (DGE) between the SHHα subtype of patients and the remaining 3 SHH subtypes (Supplemental List 2 and ref. 5) — were significantly upregulated in the SHHα subgroup in the in silico analysis of 223 human SHH MB samples by the R2 platform (http://hgserver1.amc.nl/cgi-bin/r2/main.cgi) and were also among the top-ranking genes upregulated in HN tumors, as validated by quantitative PCR (qPCR) analysis (Figure 2D).

Interestingly, the protein abundance of Yap1, N-Myc, and Gli2, which are associated with SHH-*TP53*mut and SHHα MB (5), was significantly increased in LC/A HN MB (Figure 2, E and F), with Yap1 also found in cells hyperactivating pS6 (Figure 2, E and F, and Supplemental Figure 2C). Thus, LC/A HN MB phenotypically reproduce many molecular features qualifying human LC/A SHH-*TP53*mut MB.

### The expression of the negative mTORC1 regulator Tsc2 is significantly decreased in HN MB and is regulated by p53.

**Figure 3:**
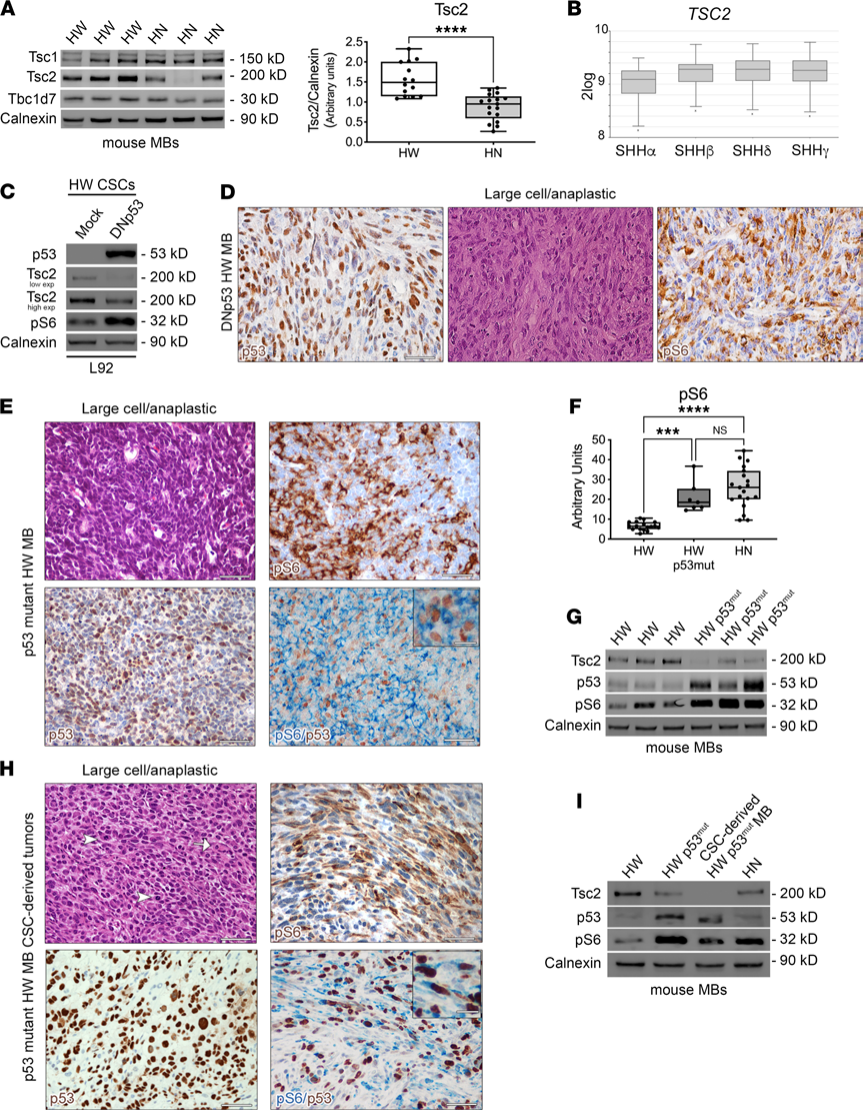
The expression of the negative mTORC1 regulator Tsc2 is significantly decreased in *HN* MB and is regulated by p53. (**A**) Protein level of Tsc1, Tsc2, and Tbc1d7 in autochthonous MB (Western blot). Densitometric quantification of Tsc2 in HW (*n* = 14) and HN MB (*n* = 17). (**B**) TSC2 expression is low in the human SHHα subtype (SHHα versus SHHβ: 1.44 × 10^–3^; SHHα versus SHHδ: 7.32 × 10^–5^; SHHα versus SHHγ: 3.3 × 10^–3^; Welch t test). (**C**) DNp53 HW CSCs show decreased Tsc2 and enhanced pS6 levels. (**D**) Tumors from DNp53 HW CSCs show high expression of the functionally inactive WT p53 (nuclear staining, brown), LC/A histology, and pS6 hyperactivation (cytoplasmic staining, blue). Scale bars: 50 μm. (**E**) LC/A features are found in HW MB with spontaneous p53 mutations (nuclear staining, brown) and correlate with the presence of pS6-IR cells that often colabeled with p53. Scale bars: 50 μm; insets: 10 μm. (**F**) Quantification of pS6 staining indicates that mTORC1 is hyperactivated in p53 mutant HW MB. (**G**) Tsc2 levels in p53 mutant HW MB are significantly lower than in HW MB, while pS6 levels are increased, as in HN MB. (**H**) Anaplastic features and p53-IR cells are detected in tumors derived from a p53 mutant HW CSC line (L68). Many pS6-IR tumor cells are p53-IR. Scale bars: 50 μm; insets: 10 μm. (**I**) Tsc2 levels in L68 MB are significantly lower than in HW MB, with pS6 levels being increased. Quantitative data are represented as a box-and-whisker plot, with bounds from 25th to 75th percentile, median line, and whiskers ranging from minimum to maximum values. Student’s *t* test, unpaired (**A**), and 1-way ANOVA followed by Tukey’s multiple comparison test (**F**). ***P < 0.005; ****P < 0.001.

To dissect the mechanisms responsible for mTORC1 hyperactivation in LC/A HN MB, we compared desmoplastic/classic HW and LC/A HN MB for the expression of (a) Tsc1, Tsc2, and Tbc1d7 that are components of the mTORC1 negative upstream regulator Tuberous Sclerosis Complex, and (b) the negative mTORC1 mediators pRaptor_S792_, Grb10, and Pten. Whereas no significant differences were observed in the expression of Tsc1, Tbc1d7 (Figure 3A), pRaptor, Grb10, and Pten (data not shown), the protein expression of Tsc2 was significantly reduced in LC/A HN MB as compared with HW MB (Figure 3A). Accordingly, in silico analysis of human SHH MB samples showed significantly reduced *TSC2* expression in SHHα specimens (Figure 3B).

To define the molecular underpinnings for Tsc2 downregulation in HN MB, we focused our attention on p53. In fact, *Tsc2* expression in cell lines and normal tissues can be directly regulated transcriptionally by p53 (29). To test if this holds true also in MB, we took advantage of p53-proficient nontumorigenic HW CSCs that we previously isolated from HW MB by the Neurosphere Assay (26). Mouse HW CSCs were transduced either with a retroviral vector (RV) coding for the GFP or for a dominant negative form of p53 (DNp53; ref. 17). The observed increased endogenous levels of p53 — which, nonetheless, is functionally inactive — are due to WT protein stabilization induced by the binding with DNp53 (30). Tsc2 expression was substantially downregulated upon p53 inactivation, and this was paralleled by a marked increase in pS6 activation (Figure 3C), suggesting that p53 mutations in MB might promote mTORC1 activation through negative regulation of Tsc2 levels. Most relevantly, p53 inactivation conferred tumorigenic ability to DNp53 HW CSCs, which gave rise to tumors showing anaplastic features and concomitant pS6 hyperactivation (6 of 10 transplanted mice with 60% penetrance; Figure 3D).

In support of the role of p53 in activating mTORC1 through Tsc2 regulation, while analyzing our cohort of HW MB, we detected a small subset of tumors (*n* = 5 of 57) that was characterized by LC/A histology (Figure 3E), in place of the desmoplastic/classic histology expected in this type of MB (Figure 1A). Interestingly, LC/A areas were characterized by the presence of many pS6-IR cells (Figure 3E). In full agreement with a previous report (31), all these outliers were endowed with the presence of more than 80% of tumor cells displaying intense nuclear staining for p53, suggestive of spontaneously occurring inactivating mutations in p53, with most pS6-IR cells being also p53-IR (Figure 3, E and F). By performing genomic DNA next-generation sequencing (NGS) of the full coding frame of p53 on 3 of these outliers, we confirmed the presence of pathogenic heterozygous somatic p53 mutations, such as C132F, Y233C, and C173G (Supplemental Figure 3). To be noted, Tsc2 expression in these p53 mutant HW MB was significantly lower than in normal HW MB (Figure 3G), in line with the observations in HN MB (Figure 3A).

Confirming these findings, a CSC line (L68) that we established from one of the HW outliers with p53 mutation acquired tumorigenic potential in vivo (ref. 32; 9 of 12 transplanted mice with 75% penetrance), as opposed to normal HW CSC lines that are devoid of this ability (25, 32). Tumors generated from this CSC line showed anaplastic traits and were characterized by the presence of many tumor cells that were IR for pS6 and, in most cases, colabeled with p53 (Figure 3H). The expression of Tsc2 was again markedly decreased in tumors derived from L68 CSC line (Figure 3I), further corroborating p53 role in the regulation of mTORC1 activation via Tsc2.

### HN MB-derived CSCs give rise to LC/A MB that hyperactivate mTORC1.

To assess the endogenous mTORC1 activation in mouse CSCs isolated from HN MB and in the tumors derived from them, we transplanted different GFP-transduced CSC lines s.c. (26, 33) into immunocompromised mice. Since HW CSCs are not tumorigenic (25, 26), as reference CSC lines for HN CSCs, we used CSCs isolated from *Ptch1*^+/–^ *p53*^+/–^ (heterozygous/heterozygous [HH]) mice that are endowed with tumorigenic potential (26). In addition to HH and HN CSC lines, we also established Myc CSC lines from Group 3 Myc–driven intracranial MB that we generated by injecting p53-null P7 cerebellar neural stem cells (NSCs; ref. 26) transduced with a RV coding for the constitutively active form of Myc, *Myc^T58A^* (17).

**Figure 4:**
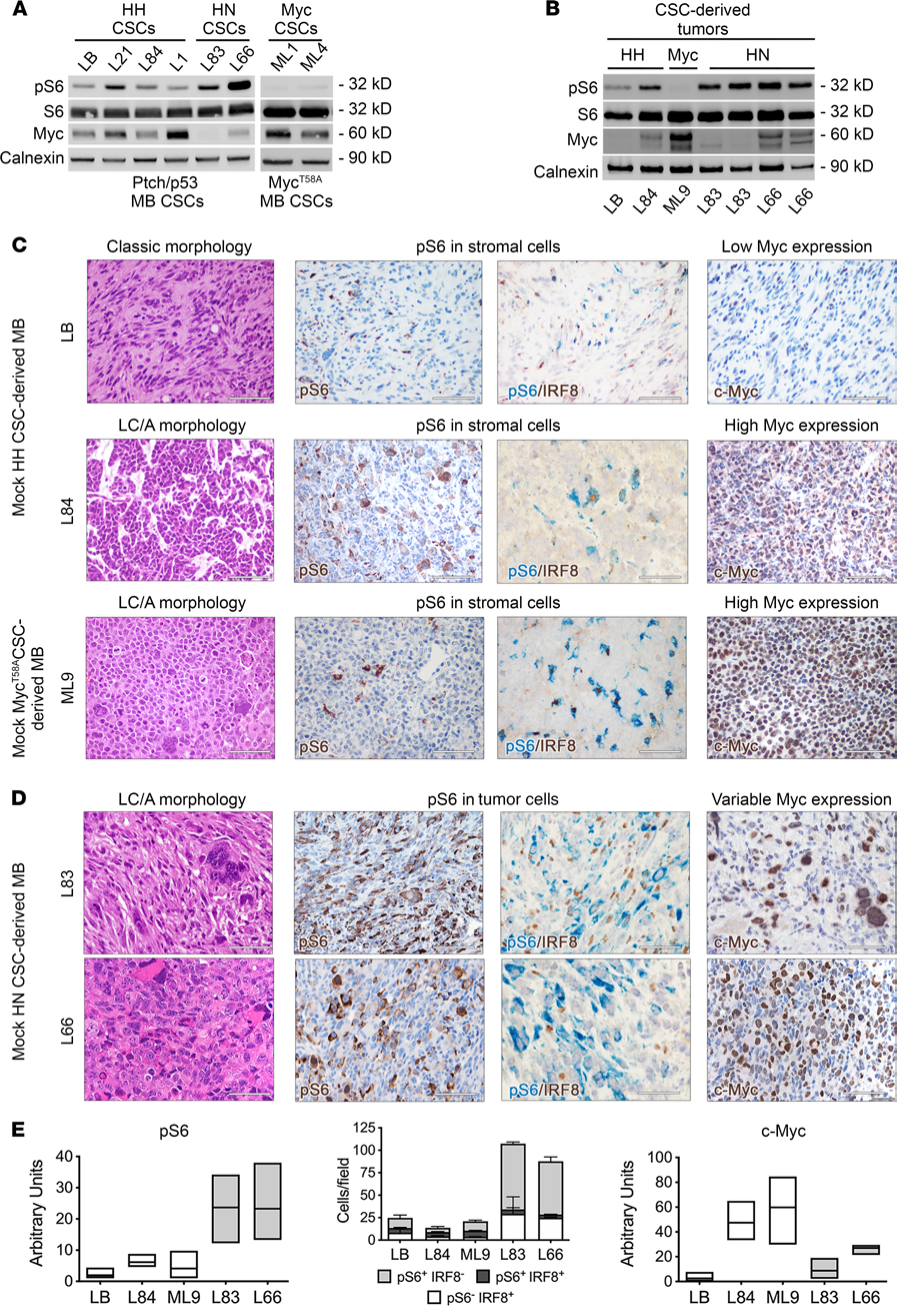
*HN* MB-derived CSCs give rise to *LC/A* MB that hyperactivate mTORC1. (**A**) Western blot analysis shows significantly higher pS6 activation and lower Myc expression in HN CSC lines than in HH and Myc^T58A^ CSC lines. (**B**) Western blot analysis shows significantly higher pS6 activation and, on average, lower Myc expression in tumors derived from the implantation of HN CSC lines compared with those from HH and Myc^T58A^ CSCs. (**C**) H&E staining showing classic features in HH CSC–derived MB, such as small cells intermingled with extensive neuropil (CSC line LB). Tumors from a subset of HH (CSC line L84) and from Myc^T58A^ CSC lines (CSC line ML9) are endowed with LC/A characteristics, such as nuclear molding, prominent nucleoli, and apoptosis; in both cases, pS6-IR cells are few (cytoplasmic staining, brown) and are double-labeled with IRF8, indicating that they are tumor stromal cells (pS6, cytoplasmic staining, blue; IRF8, nuclear staining, brown). c-Myc (nuclear staining, brown) is lowly expressed in classic HH CSC–derived tumors, whereas it is very highly expressed in LC/A tumors derived from a subset of HH (CSC line L84) and from Myc^T58A^ CSC lines (CSC line ML9). All scale bars: 50 μm. (**D**) Tumors from HN CSCs (CSC lines L83 and L66) show LC/A traits, such as increased nuclear pleomorphism, presence of large cells, and high level of cellular atypia. pS6 is hyperactivated in tumor cells (cytoplasmic staining, brown), as demonstrated by the absence of colabeling of pS6^+^ cells (cytoplasmic staining, blue) with IRF8 (nuclear staining, brown). c-Myc expression is found in medium-to-high number of cells. All scale bars: 50 μm. (**E**) Quantification of the level of marker expression is shown in the graphs. Quantitative data are presented as floating bars from minimum to maximum values, line at mean. One-way ANOVA followed by Tukey’s multiple comparison test. See Supplemental Table 5 for detailed statistical analysis.

HH CSC lines (LB, L21, L84, and L1) did activate mTORC1 at low level, while expressing variable levels of Myc (Figure 4A). Similarly, *Myc^T58A^* CSC lines (ML), such as ML1 and ML4, had constitutively high Myc expression but did not hyperactivate pS6. On the contrary, HN CSC lines, such as L83 and L66, showed a remarkably high activation of pS6, with low levels of Myc (Figure 4A).

In line with in vitro findings, tumors derived from the implantation of HH CSCs (LB and L21) showed “classic” morphology (26), low Myc expression, and mTORC1 activation that was solely observed in IRF8^+^ macrophages/microglia cells (Figure 4, B and C). Tumors derived from a small subset of HH CSC lines (L84) were characterized by LC/A features and high Myc protein level; however, mTORC1 activation was never detected in tumor cells, while being again confined to the stromal compartment. Likewise, MB originating from Myc CSC lines were LC/A with constitutive Myc activation, but again, they showed mTORC1 activation only in stromal cells (Figure 4, B, C, and E, and Supplemental Table 5). Most notably, MB induced by HN CSC lines (i.e., L66 and L83) displayed LC/A features associated with significantly high mTORC1 activation specifically in tumor cells (Figure 4, B, D, and E, and Supplemental Table 5), thereby recapitulating autochthonous HN tumors. Thus, also in experimental tumors induced from the implantation of mouse CSCs, LC/A morphology correlated with increased mTORC1 activation.

### Enforced hyperactivation of mTORC1 in HH/HN CSCs increases tumor malignancy, induces an LC/A phenotype, and modulates the expression of subgroup-restricted markers.

**Figure 5:**
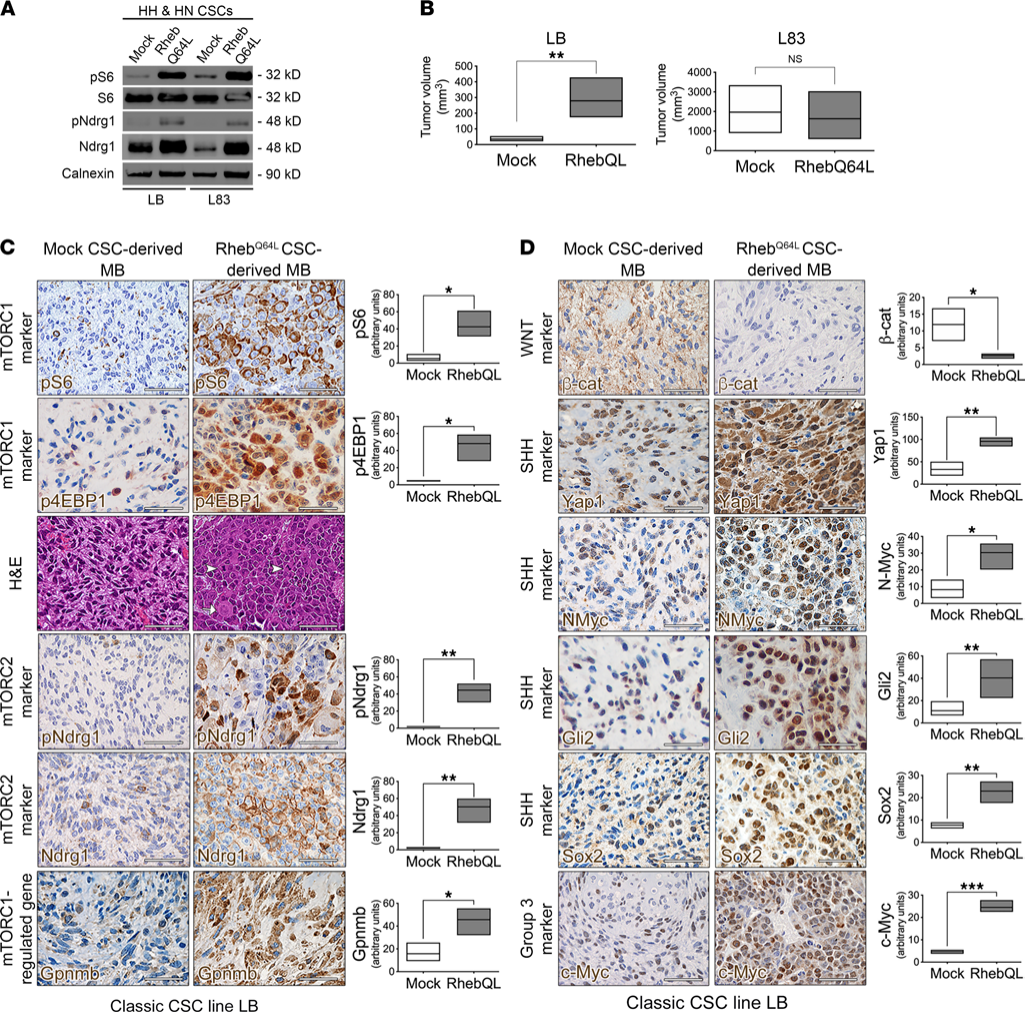
Enforced hyperactivation of mTORC1 in *HH*/*HN* CSCs increases tumor malignancy, induces an *LC/A* phenotype, and regulates MB subgroup specification by modulating the expression of subgroup-restricted markers. (**A**) Western blot showing that mTORC1 hyperactivation in HH/HN *Rheb^Q64L^* CSC lines also promotes the activation of mTORC2 (HH CSC line LB and HN CSC line L83). (**B**) Hyperactivation of *Rheb^Q64L^* in classic HH CSCs (LB) give rise to tumors that grow faster and larger than controls (volume measured at 54 days after transplant for LB). The same hyperactivation in LC/A HN CSCs (L83) does not significantly affect the rate of tumorigenesis (volume measured at 83 days after transplant for L83). (**C**) H&E staining showing pS6 and p4EBP1 hyperactivation in tumor cells in MB derived from classic HH CSCs (LB) after transduction with *Rheb^Q64L^*. HH *Rheb^Q64L^* MB are endowed with typical LC/A features— e.g., nuclear molding, large cells (white arrow) and several mitotic figures (white arrowheads). The mTORC2 marker pNdrg1 is also strongly hyperactivated in HH *Rheb^Q64L^* MB. The mTORC1 regulated gene Gpnmb is highly expressed in *Rheb^Q64L^* MB. All scale bars: 50 μm. Quantification of the level of marker expression (shown as arbitrary units) is shown in the graphs (right panels). (**D**) The WNT-associated classifier β-catenin is significantly downregulated in *Rheb^Q64L^* HH CSC-derived MB (CSC line LB), whereas markers typical of p53 mutant SHH MB, such as Yap1, N-Myc, Gli2, and Sox2, are upregulated. The Group 3 classifier c-Myc is also overexpressed in *Rheb^Q64L^* HH CSC–derived MB. All scale bars: 50 μm. Quantification of the level of marker expression (shown as arbitrary units) is shown in the graphs (right panels). Quantitative data are presented as floating bars from minimum to maximum values, line at mean. Student’s *t* test, unpaired. See Supplemental Table 5 for detailed statistical analysis. *P < 0.05; **P < 0.01; ***P < 0.005.

To prove that mTORC1 hyperactivation was causally linked to the acquisition of LC/A features, we transduced both classic HH CSCs (LB and L21) and, as a control, LC/A HN CSCs (L83) with a lentiviral vector (LV) coding for the constitutively active form of the mTORC1 activator Rheb, *Rheb^Q64L^* (34), or with a mock vector coding for GFP. Both classic and LC/A *Rheb^Q64L^*–transduced CSCs showed significant hyperactivation of pS6, when compared with controls (Figure 5A, Supplemental Figure 4A, and Supplemental Figure 5). Interestingly, all *Rheb^Q64L^* CSC lines showed increased expression and phosphorylation of NDRG1, the latter suggestive of concomitant mTORC2 hyperactivation (Figure 5A, Supplemental Figure 4A, and Supplemental Figure 5).

*Rheb^Q64L^* HH CSC–derived tumors developed in a shorter time frame and grew larger than controls (Figure 5B and Supplemental Figure 4B), whereas no difference in growth was observed in *Rheb^Q64L^* LC/A HN CSC–derived tumors with respect to mock (Figure 5B).

Most significantly, as opposed to the classic morphological features observed in GFP-transduced HH CSC-derived tumors (26), *Rheb^Q64L^* HH CSC–derived tumors, which comprised many cells hyperactivating both pS6 and p4EBP1, acquired de novo typical features of LC/A tumors, such as the presence of large cells, nuclear molding, anaplastic traits, high mitotic activity, and increased apoptosis (Figure 5C, Supplemental Figure 4C, and Supplemental Table 1). Indeed, almost all pS6-IR cells found in *Rheb^Q64L^* HH CSC–derived tumors were large and showed typical morphological LC/A features, such as the presence of angular nuclei and prominent nucleoli (Figure 5C and Supplemental Figure 4C). Like LC/A autochthonous HN MB, *Rheb^Q64L^* HH CSC–derived tumors did show increased activation of mTORC2, as well as enhanced expression of Gpnmb (Figure 5C and Supplemental Figure 4C).

After transduction with *Rheb^Q64L^*, spontaneous LC/A HN CSCs (L83) gave rise to tumors that acquired additional LC/A features, such as the presence of large cells with multiple nucleoli, characterized by nuclear molding (Supplemental Table 1), as well as increased pS6, p4EBP1, pNDRG1, and Gpnmb expression (Supplemental Figure 5A).

Concerning tumor subgroup affiliation, hyperactivation of mTORC1 via *Rheb^Q64L^* in classic HH CSCs resulted in the significant downregulation of WNT pathway–specific markers such as β-catenin (Figure 5D and Supplemental Figure 4C) with concurrent upregulation of p53 mutant SHH subgroup–specific markers, such as Yap1, N-Myc, Gli2, and Sox2 (Figure 5D and Supplemental Figure 4C). Also, expression of c-Myc was enhanced in *Rheb^Q64L^* HH CSC–derived tumors, as observed in autochthonous HN MB (Figure 5D and Supplemental Figure 4C). Similar findings were observed in LC/A HN CSCs after transduction with *Rheb^Q64L^*, which showed significantly enhanced mTORC2 hyperactivation and increased Gpnmb expression (Supplemental Figure 5A). Although most subgroup markers were already upregulated in mock HN CSC–derived tumors, a significant difference was noticeable for Gli2, Sox2, and c-Myc (Supplemental Figure 5B).

When the same classic mock and *Rheb^Q64L^* HH CSCs (LB) were injected intracranially (Supplemental Figure 6), mTORC1-hyperactivating tumors again showed highly malignant histological features and developed in a very short time window (53 ± 14 days for *Rheb^Q64L^* tumors versus 197 ± 18 days for *GFP* tumors). As opposed to mock tumors showing classic histological features, *Rheb^Q64L^*-transduced tumors acquired typical LC/A traits, with diffuse anaplastic areas and regions containing cells with nuclear molding (Supplemental Figure 6). As such, mTORC1 hyperactivation directly promotes the acquisition of highly malignant LC/A characteristics in MB.

### Pharmacological targeting of the mTOR pathway hampers the growth of autochthonous SHH-TP53mut MB.

**Figure 6:**
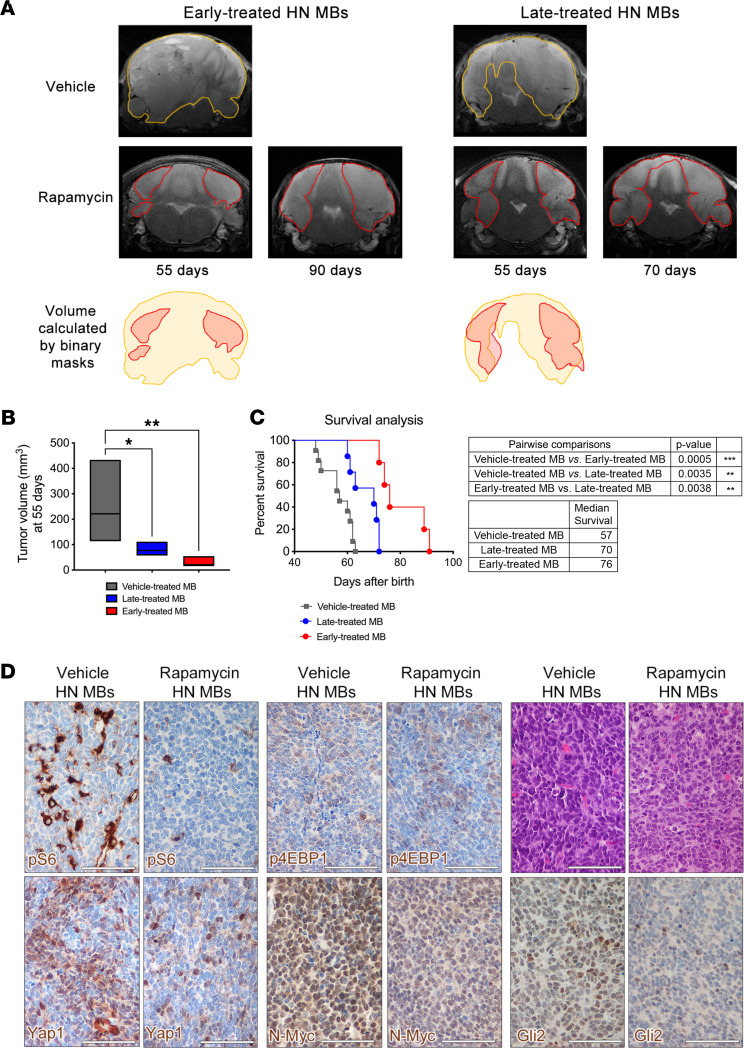
Pharmacological targeting of the mTOR pathway hampers the growth of autochthonous SHH-TP53 mutant MB. (**A**) Longitudinal T2-weighted MRI analysis indicates that rapamycin administration significantly impairs the growth of early- and late-stage HN MB. (**B**) MB volume, as calculated by binary masks at day 55 of age — i.e., the MRI time point closer to the median survival of control mice — is significantly reduced by both rapamycin treatment regimens when compared with vehicle-treated controls (late-treated HN MB, *P <0.05; early-treated HN MB, **P <0.01). At the latest time points assessed for early (90 days) and late-treated (70 days) HN MB, both treated HN MB are significantly smaller than controls at 55 days. Statistical analysis is shown in the upper right table. (**C**) Kaplan-Meier survival curves show that the lifespan of HN mice treated with rapamycin at early and late stages of tumor development is significantly increased as compared with control mice (*n* = 11 for vehicle-treated mice, *n* = 5 for early-treated mice, *n* = 7 for late-treated mice). (**D**) Following late rapamycin treatment, HN MB show a significant reduction in the activation of pS6, but not of p4EBP1, in nuclear pleomorphism and molding, as well as in Yap1, N-Myc, and Gli2 expression. All scale bars: 50 μm. See Supplemental Figure 8 for detailed statistical analysis. Quantitative data are presented as floating bars from minimum to maximum values, line at mean. One-way ANOVA, followed by Dunnett’s multiple comparison test (**B**), and log rank test (**C**). *P < 0.05; **P < 0.01.

To test whether increased mTORC1 activation in LC/A MB could give rise to a potentially targetable vulnerability in SHH-*TP53*mut human MB, we designed a preclinical Phase 2–like trial in which different types of autochthonous mouse MB were treated with the brain-permeant mTOR inhibitor rapamycin (6 mg/kg) for 5 days a week for up to 80 days of treatment (Figure 6 and Supplemental Figures 7 and 8).

To mimic both lowly and highly severe clinical settings (i.e., the presence of either minimal residual disease/small tumors or large unresectable tumors), we set up a randomized 3-arm interventional trial by treating LC/A HN mice at early and late stages of tumor development and by longitudinally monitoring tumor growth by 7-Tesla conventional T2-weighted MRI. Early-treated HN mice started rapamycin between 10 and 12 days of age, whereas late-treated HN mice began treatment at 23–27 days of age. As a negative control, we treated classic HW mice, which activate mTORC1 only in stromal cells and, thus, should not respond to treatment.

In fact, HW mice treated with rapamycin showed only a slight reduction in tumor volume (Supplemental Figure 7, A and B). Conversely, a statistically significant decrease in tumorigenesis was detected in rapamycin-treated HN mice bearing either small or large tumors, as compared with vehicle-treated controls, with a significantly higher reduction in volume in early-treated compared with late-treated HN mice (Figure 6, A and B, and Supplemental Figure 7, C and D). Tumor response to treatment was associated with a 34% and 23% increase in median survival of early- and late-treated HN mice, respectively (Figure 6C).

IHC analysis demonstrated that activation of the mTORC1 downstream surrogate marker pS6 was downregulated in HN tumors following rapamycin delivery in vivo, suggesting effective target engagement during treatment (Figure 6D and Supplemental Figure 8, A and C). On the contrary, phosphorylation of 4EBP1 on Thr37/46, which is known to be rapamycin insensitive, was unaffected by the treatment (Figure 6D and Supplemental Figure 8A). mTOR inhibition induced changes in HN MB morphology, with a significant decrease in nuclear molding and pleomorphism, as well as an overall reduction in anaplastic traits (Figure 6D). Expression of Yap1, N-Myc, and Gli2 was also reduced by treatment (Figure 6D and Supplemental Figure 8B).

### Pharmacological targeting of the mTOR pathway significantly impairs the growth of CSC-derived mTOR–driven LC/A MB but not that of MB belonging to other subgroups.

To capture the intertumor molecular heterogeneity of a MB patient population, we designed a master interventional preclinical trial by treating with rapamycin several molecularly different mouse CSC–derived MB. These tumors were proxies of (a) spontaneously mTORC1-activating LC/A SHH-*TP53*mut MB, (b) enforced mTORC1-activating *Rheb^Q64L^*-transduced LC/A SHH-*TP53*mut MB, (c) enforced mTORC1 activating *Rheb^Q64L^*-transduced classic SHH-*TP53*mut MB, (d) classic WNT-like MB (26), (e) Myc-driven LC/A MB, and (f) *Myc^T58A^*-transduced LC/A CSC MB. We transplanted all the different types of CSCs into the flank of immunocompromised mice and allowed tumors to become palpable before performing randomization and starting rapamycin treatment.

**Figure 7:**
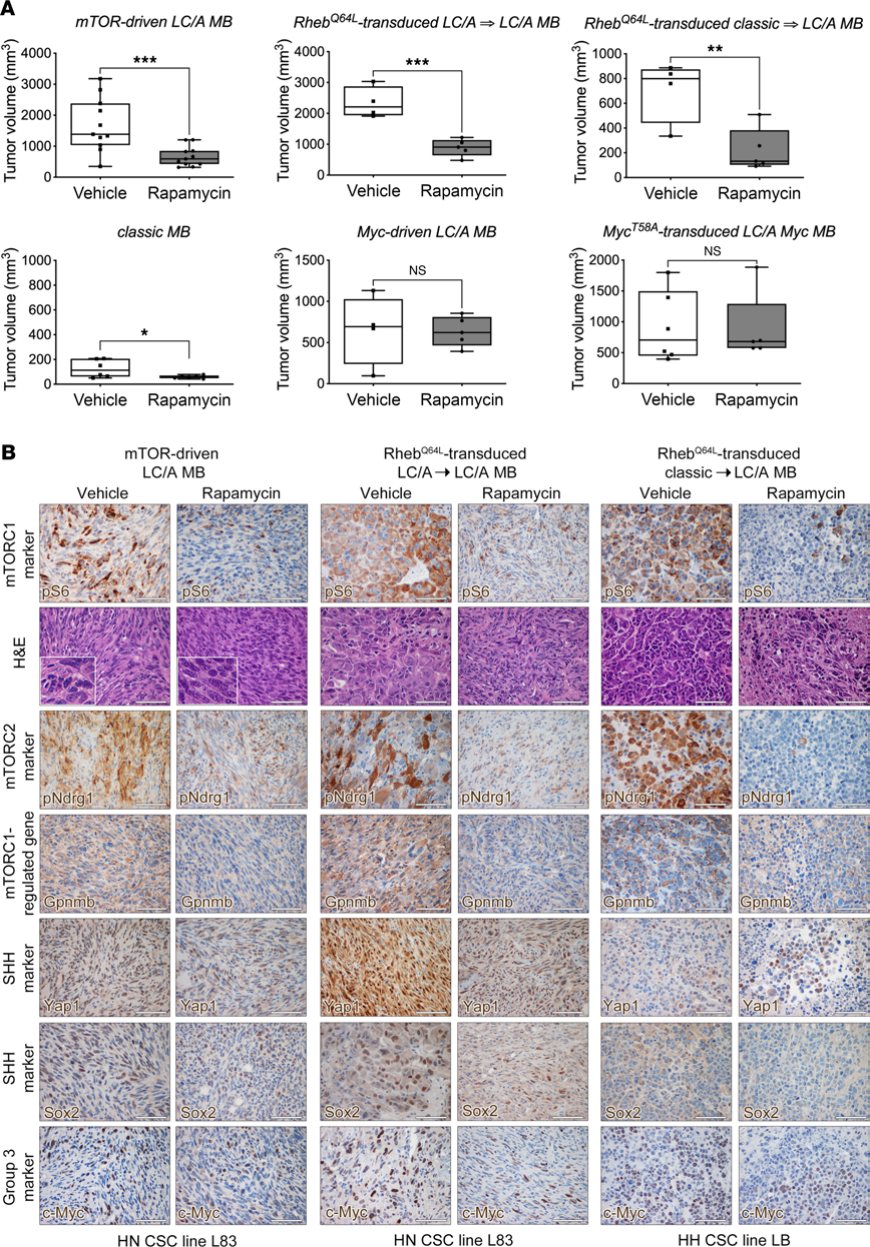
Pharmacological targeting of the mTOR pathway significantly impairs the growth of CSC-derived mTOR–driven *LC/A* MB but not that of MB belonging to other subgroups. (**A**) Tumor growth is significantly reduced by rapamycin treatment in LC/A tumors generated by HN CSCs with endogenous activation of mTORC1 (L83 and L66, 28 days of treatment for both vehicle and rapamycin arms; *n* = 11), the same CSCs transduced with *Rheb^Q64L^* (L83, 25 days of treatment for both vehicle and rapamycin arms; *n* = 5), and classic HH CSCs transduced with *Rheb^Q64L^* (17 days of treatment for vehicle arm; 30 days of treatment for rapamycin arm; *n* = 6). Conversely, tumor growth was only slightly affected by rapamycin treatment in classic tumors generated by classic HH CSCs (LB, 30 days of treatment for both vehicle and rapamycin arms; *n* = 6). No significant differences in tumor volume were detected in LC/A tumors induced by HH CSCs with no endogenous hyperactivation of mTORC1 (L84, 40 days of treatment for both vehicle and rapamycin arms; *n* = 5) and *Myc^T58A^*-transduced CSCs (ML9, 20 days of treatment for both vehicle and rapamycin arms; *n* = 6). (**B**) H&E analysis following rapamycin treatment of the different types of LC/A MB shows a significant reduction in nuclear pleomorphism (insets in mTOR-driven LC/A MB, left panels), in nuclear molding and in cell size (middle and right panels). Activation of both pS6 and pNdrg1 was strongly decreased by treatment, and so was the expression of Gpnmb, Yap1, and Sox2. All scale bars: 50 μm. Quantitative data are represented as a box-and-whisker plot, with bounds from 25th to 75th percentile, median line, and whiskers ranging from minimum to maximum values. Student’s *t* test, unpaired. *P < 0.05; **P < 0.01; ***P < 0.005.

In line with findings obtained in autochthonous HN MB, rapamycin administration resulted in the significant reduction in growth of spontaneous LC/A and enforced LC/A *Rheb^Q64L^* HN and HH CSC–derived tumors, but not of classic WNT, Myc-driven LC/A, and Group 3 Myc MB (Figure 7A).

mTOR inhibition promoted a substantial reduction in nuclear pleomorphism and cell size, as well as decreased nuclear molding in the presence of an enlarged intercellular matrix (Figure 7B). As expected, protracted rapamycin treatment impaired not only mTORC1, but also mTORC2 activation (Figure 7B and Supplemental Figure 9). mTORC1 inhibition also induced downregulation of Gpnmb (Figure 7B and Supplemental Figure 9). Expression of Yap1 and Sox2 was slightly reduced, whereas no significant difference in expression was observed for c-Myc (Figure 7B and Supplemental Figure 9). Collectively, these data indicate that inhibition of mTORC1 signaling may lead to a significant improvement in disease control in SHH-*TP53*mut MB as compared with the other MB subgroups.

### mTORC1 activation is specifically found in human p53 mutant SHH MB with a LC/A component and may be a subgroup-specific therapeutic vulnerability.

To determine whether mTORC1 hyperactivation could be detected in human LC/A SHH-*TP53*mut MB, thus potentially serving as diagnostic marker for this specific cohort of patients, we performed IHC for pS6 on 90 human MB specimens, comprising all the distinct molecular subgroups of MB. SHH-*TP53*mut MB represent 21% of SHH MB that account for 29% of all MB (10). Considering that only 40% of SHH-*TP53*mut MB show LC/A histology, the frequency of patients with LC/A SHH-*TP53*mut MB is very low (3% of all MB; ref. 10). As such, we were able to collect 8 specimens of LC/A SHH-*TP53*mut MB from 2 different institutions (Spedali Civili and University of Brescia, Brescia, and Istituto Neurologico Besta, Milan, Italy). As controls, we analyzed 15 desmoplastic/classic SHH-*TP53* WT MB, 4 classic WNT MB, 51 classic/desmoplastic non-WNT/non-SHH MB, and 12 LC/A non-WNT/non-SHH MB (Supplemental Table 6).

**Figure 8:**
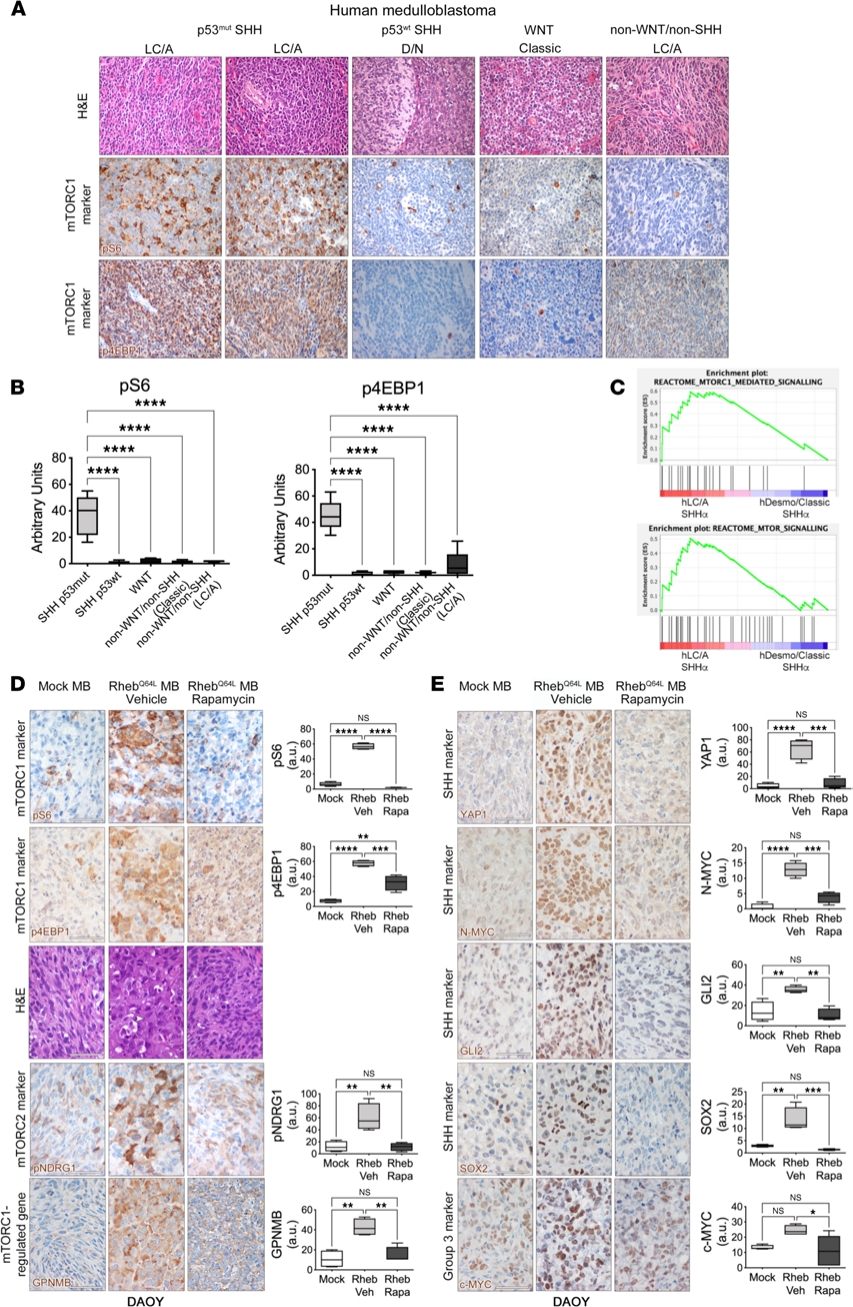
mTORC1 activation is specifically found in human p53 mutant SHH MB with *LC/A* component and may be a subgroup-specific therapeutic vulnerability. (**A**) High numbers of tumor cells positive for the mTORC1 surrogate markers pS6 and p4EBP1 (cytoplasmic staining, brown) are observed only in human LC/A MB belonging to the SHH subgroup with p53 mutation and are not detected in human desmoplastic/nodular (D/N) SHH p53^wt^, classic WNT, and LC/A non-WNT/non-SHH MB. Scale bars: 50 μm. (**B**) Quantification of pS6 and p4EBP1 expression in the different subgroups. Only statistically significant pairwise comparisons are shown. (**C**) GSEA indicates that the genes qualifying ‘human p53 mutant SHHα MBs with LC/A histology’ are significantly enriched in two different mTOR-related gene signatures. (**D**) Tumors from human DAOY cells after *Rheb^Q64L^* transduction show increased frequency of pS6- and p4EBP1-IR tumor cells, which are very few in mock tumors and are reduced in number after treatment with rapamycin. *Rheb^Q64L^* transduction promotes the acquisition of LC/A traits, such as the presence of large cells with prominent nucleoli (white arrowheads), which are not observed in controls and are strongly diminished by rapamycin (H&E). The activation of pNDRG1 and the expression of GPNMB are both increased in *Rheb^Q64L^*-transduced MB and turned off by rapamycin. Scale bars: 50 μm. Quantification of the level of marker expression is shown in the graphs. (**E**) The expression of YAP1, N-MYC, GLI2, SOX2, and c-MYC is enhanced by mTORC1 hyperactivation and decreased by rapamycin administration. Scale bars: 50 μm. Quantification of the level of marker expression is shown in the graphs. Quantitative data are represented as a box-and-whisker plot, with bounds from 25th to 75th percentile, median line, and whiskers ranging from minimum to maximum values. One-way ANOVA followed by Tukey’s multiple comparison test. *P < 0.05; **P < 0.01; ***P < 0.005; ****P < 0.001.

The same correlation between the presence of LC/A features and mTORC1 hyperactivation observed in mouse preclinical models was detected only in human SHH-*TP53*mut MB specimens, which all showed high frequency of pS6- and p4EBP1-IR tumor cells (Figure 8, A and B). Conversely, samples from all the other subgroups never hyperactivated pS6 in tumor cells — only rarely in stromal cells (Figure 8, A and B, and Supplemental Table 6). N-MYC, GLI2, and YAP1 proteins were detected also in other molecular subgroups (Supplemental Figure 10, A and B); if considering only SHH MB, all 3 proteins were significantly upregulated in SHH-*TP53*mut MB as compared with SHH-*TP53* WT MB (Supplemental Figure 10C). Thus, mTORC1 activation is associated with LC/A histology only in human SHH-*TP53*mut MB.

To further confirm this finding, we subjected the genes differentially expressed between human SHHα MB with LC/A histology versus human SHHα MB with desmoplastic/classic histology to GSEA (5) with human transcriptional data sets containing mTOR-related signatures (Figure 8C); we observed that statistically significant enrichment for mTOR- and mTORC1-specific genes was found in human SHHα MB that were characterized by LC/A histology and, for 75% of them, also by p53 mutation (Supplemental Table 7).

To explore the functional relevance of mTORC1 activation in human SHH-*TP53*mut MB, we took advantage of the human primary MB cell line DAOY. Although being classified as a SHH MB cell line with p53 mutation (35), the DAOY cell line was originally established from a desmoplastic MB (36). Since desmoplastic SHH MB very rarely show p53 mutations (14), it is likely that DAOY cells acquired p53 mutations after culturing in vitro. Accordingly, in vivo implantation of naive DAOY cells gave rise to MB that did not show mTORC1 activation in tumor cells and were endowed with desmoplastic/classic histology, implying that p53 mutations should be present in the original tumor to be able to influence the molecular make-up of the tumor (Figure 8D and Supplemental Table 1). DAOY cells were transduced with the *Rheb^Q64L^* LV, and as observed in mouse MB CSCs, mTORC1 hyperactivation increased the expression of NDRG1 and its phosphorylation (Supplemental Figure 11A). *Rheb^Q64L^* DAOY cells gave rise to tumors that grew faster and were more malignant than controls (Supplemental Figure 11B). These tumors showed acquisition of some LC/A features (i.e., the presence of large cells with multiple nucleoli, increased mitotic figures, and apoptotic cells), hyperactivated mTORC2 (Figure 8D and Supplemental Table 1), and increased expression of GPNMB, YAP1, N-MYC, GLI2, SOX2, and c-MYC (Figure 8, D and E). Thus, enforced mTORC1 activation in DAOY cells may give rise to a reliable human preclinical model of LC/A SHH–*TP53*mut MB. Notably, this model is also characterized by increased expression of N-MYC and GLI2, as expected in N-MYC/GLI2 amplified SHH-*TP53*mut MB.

Finally, we took advantage of this human MB model to test whether mTORC1 inhibition could reduce the growth of human LC/A SHH p53 mutant tumors in vivo. A 3-fold reduction in tumor volume was observed in LC/A *Rheb^Q64L^* DAOY–derived MB treated with rapamycin as compared with vehicle-treated controls, while no difference was observed between vehicle- and rapamycin-treated classic *GFP* DAOY–derived MB (Supplemental Figure 11C). Rapamycin treatment inhibited mTOR activation, as shown by a significant decrease in both pS6 and pNDRG1 and by only a mild reduction in p4EBP1 (Figure 8D). A significant decrease in large cells and anaplastic traits, such as nuclear pleomorphism and mitotic figures (Figure 8D), and in the levels of GPNMB, YAP1, N-MYC, GLI2, SOX2, and c-MYC was observed after mTORC1 inhibition (Figure 8, D and E).

## Discussion

In recent years, no efforts were spared to improve the identification of the molecular players underlying MB development and evolution. Different molecular subgroups and subtypes of the disease have been identified that are endowed with specific genomic, epigenomic, transcriptomic, and proteomic profiles (37). This wealth of knowledge is currently used to inform the development of potentially novel therapeutic avenues that take into consideration the molecular status of MB, aiming at implementing molecularly targeted clinical protocols relying on patient stratification and selection (37).

In line with this view, most preclinical studies are now focusing on the definition of the molecular makeup of MB, to identify meaningful molecularly targeted approaches to be translated into the clinical settings. Within this frame of mind, we reasoned that already available mouse models of MB might be instrumental to this aim, if characterized in greater detail both from a morphological and molecular perspective.

In this study, we provided a comprehensive investigation of one on the most widely used mouse models of SHH MB — i.e., the *Ptch*^+/–^ mouse model with or without concurrent mutation in *TP53* (19) — by subjecting to histomorphological analysis large cohorts of mice with different genotypes. By this approach, for the first time to our knowledge, we report that *Ptch1*^+/–^ *p53*^–/–^ (HN) mice show distinctive features of LC/A MB, which are not observed in *Ptch1*^+/–^ *p53*^+/+^ (HW).

Although concurrent loss of *PTCH1* and *TP53* has been found in a few LC/A SHHα human MB (15), our model might not completely reproduce the genetics of the disease, which in most cases is known to be associated with amplification in *MYCN* and *GLI2* (14, 37, 38). While it is desirable that a preclinical model mimics all the features of the corresponding human tumor in terms of genetics, cell of origin, clinical manifestations, histological phenotype, and response to therapy, none of the currently available models of MB fulfill all these requirements at the same time (39). Indeed, many studies are available in which specific mutations that are not normally observed in human patients have been exploited to implement subgroup-specific models. For instance, mouse models of Myc and Wnt subgroups can be generated only after introducing p53 mutations that are very rarely detected in the corresponding tumors in patients (16–18). Nevertheless, these preclinical models are valuable phenocopies of the human disease, indicating that modeling of a disease-specific phenotype may take place also in the absence of a disease-specific genotype. In our study, by combining different experimental mouse models, such as autochthonous and CSC-derived MB, with human MB samples (human postsurgery specimens and human cell lines), we have generated a comprehensive histological, molecular, and functional experimental platform that provided sound evidence that mTOR activation is a therapeutic target in LC/A SHH–*TP53* MB, independent from the genetic mutations responsible for SHH pathway hyperactivation — e.g., upstream mutations in *PTCH1* or downstream amplification of *N-MYC* and *GLI2*. Indeed, *Ptch1*^+/–^ *p53*^–/–^ MB are characterized by upregulation of N-Myc and Gli2, as to mimic what it is supposed to take place in *N-MYC* and/or *GLI2* amplified SHH MB.

To pinpoint the molecular mechanisms underlying the malignant morphological features observed in HN MB, we focused on the mTOR pathway, which is known to control cell size and growth. mTOR signaling is hyperactive in a large proportion of human cancers, and accordingly, mutations in distinct components of the pathway have been etiologically involved in several cancers (40).

In this study, we provide evidence that the activation of mTOR is specifically associated with a subset of human SHH MB that are frequent in children and are characterized by LC/A histology and p53 mutation. The significance of mTOR hyperactivation in MB is controversial, as reviewed in refs. 20 and 22. Concerning the assessment of mTOR activation in specific preclinical mouse models of MB, activation of mTORC1, and its interaction with the SHH pathway were assessed in a *SmoM2* mouse model during normal cerebellar development and were positively associated with MB initiation (21). However, this seminal study — which paved the way to ours — while finely dissecting the interplay between mTORC1 and SHH pathways, focused entirely on p4EBP1, which is considered 1 of the 2 surrogate markers for mTORC1 activation but, notably, is also regulated by other kinases in a mTOR-independent way (41). In fact, the data on p4EBP1 activation in the different molecular subgroups of human MB reported in the Wu’s study are at odds with ours, which were obtained by using not only p4EBP1, but also the other mTORC1 surrogate marker pS6 — i.e., the gold standard in the field — and by also including the histological affiliation of the human MB samples analyzed.

Targeting mTOR has also been suggested, in a review, as a potential therapeutic strategy for Group 4 MB (22), and accordingly, mTORC1 hyperactivation has been recently reported in human cell lines genetically modified to model Group 4 MB (42). Although some studies reported the efficacy of mTOR inhibitors in different human MB cell lines in vitro (23), no detailed analysis with respect to the molecular subgroup and the persistence of mTOR activation was undertaken in vivo. Indeed, in our study, we report that the activation of mTOR in CSCs and human MB cell lines in vitro does not predict its activation in the corresponding tumor after transplantation.

As for the significance of mTOR activation specifically in human SHH MB, mTORC1 has been variably associated with different molecular subtypes of the SHH subgroup, such as infant (24) and adult SHH MB (14). As opposed to our findings, in the first study, mTOR activation — which was dependent on OCT4 expression in *MYCN*-overexpressing induced pluripotent stem cells (iPSC) — led to the formation of xenografts with clear desmoplastic traits (24). However, although mTORC1 activation by IHC for p4EBP1 was reported in iPSC-derived xenografts and in patient-derived xenografts, it was not analyzed in human postsurgery samples of infant SHH MB. As a matter of fact, in our collection of infant SHH MB (*n* = 13), all showing desmoplastic histology, we never detected mTORC1 activation in tumor cells. Thus, mTOR activation in iPSC-derived humanized preclinical models might be secondary to the genetic modifications induced in iPSC rather than being a molecular trait associated with the infant subgroup of the human disease. In the second study, mTOR activation was observed in 30% of adult MB, which are neither characterized by LC/A histology nor by p53 mutations. However, in the same study, some cases of children’s MB were also reported to be positive for pS6. It may be possible that the pS6^+^ children’s SHH MB belonged to the LC/A-p53 mutant cohort of patients, thus increasing their relative frequency and being, therefore, in agreement with our findings.

As for the mechanisms responsible for mTOR hyperactivation, we have identified a consistently reduced expression of the mTORC1 inhibitor Tsc2 in *Ptch1*^+/–^ *p53*^–/–^ MB that has been detected at the mRNA level also in SHHα MB patients. Indeed, genetic inactivation of Tsc2 protein, which results in the destabilization of the TSC complex and in the ensuing Rheb-dependent activation of mTORC1, has been shown to have a positive impact on cerebellar granule cell expansion and MB initiation (43). As for the low level of Tsc2 observed in our p53-mutant mouse models, the expression of Tsc2 might be directly regulated transcriptionally by p53 binding to its promoter (29, 44). Indeed, here we demonstrated that loss of p53 activity in p53-proficient HW MB CSCs and in a subset of HW MB outliers with spontaneous p53 mutations enhances mTORC1 activity by decreasing Tsc2 expression, thus providing first evidence to our knowledge that the p53-dependent regulation of Tsc2 may be active also in MB.

mTOR inhibitors are currently under clinical testing for many cancer types, including pediatric brain tumors. A few basket trials are ongoing or have been recently completed that included very small numbers of MB patients, with only 1 trial that recruited patients based on the presence of inactivating mutations in the PI3K/mTOR pathway, although there was no reference to the original molecular subgroup/subtype. Given the low number of MB patients and the lack of comprehensive molecular and histological information, the results of these studies might be discouraging. On the contrary, clinical studies, potentially targeting a specific and molecularly uniform subpopulation of MB patients such as the one we identified here, might generate valuable information that may directly impact treatment decisions and disease prognosis (45).

As mentioned before, although a targeted approach aiming at inhibiting mTOR activation might be life-changing for SHH-*TP53*mut MB patients, they are quite infrequent. To increase the applicability of this therapeutic paradigm, we may take into consideration the fact that patients with recurrent MB often present with LC/A histology and with *TP53* mutations that were undetected in the primary tumor (46). In fact, loss of function of *Trp53* is identified as a key event in the pathogenesis of recurrence (38, 46–48). Specifically, genetic events in *TP53* pathway genes or in the actual *TP53* gene are frequent in recurrences, predominantly in SHH MB (38, 48). Very interestingly, the mouse model used for modeling posttreatment MB recurrence, namely a *Tp53*mut transposon-driven MB model, shows large cells, nuclear atypia, and nuclear molding that are all typical features of LC/A histology (49). Accordingly, another report demonstrated that combined p53 and MYC defects emerge at MB recurrence and that relapsed SHH MB with altered p53/MYC were significantly associated with LC/A pathology (47). Likewise, metastasis through leptomeningeal dissemination has been associated with LC/A SHH MB (5, 49). Thus, testing the hyperactivation of mTOR in recurrent/metastatic MB might increase the numbers of patients who may benefit from this personalized medicine approach.

The current indications for unlocking precision medicine–based approaches in the clinical practice propose that disentangling the plethora of signaling mechanisms regulating the different variant of a tumor might require a more comprehensive understanding of smaller subsets of patients, segregated by specific molecular processes, and a deeper understanding of how these distinct cohorts relate to each other (50). This concept has been recently emphasized in the WHO CNS5, which discusses that, although it may be challenging to identify biologically defined groups that may be too small for clinical trials to be designed around, it is important to build upon the availability of subgroup/subtype-specific molecular vulnerabilities, whose clinical impact may be lost if molecularly distinct patients are treated the same way (7).

We believe that the findings here reported are in full agreement with this view, by relying (a) on strong evidence collected in several preclinical mouse models mimicking the different MB subgroups, (b) on the functional validation of the molecular mechanisms involved by using the same models, (c) on the pharmacological treatment by different experimental paradigms, (d) on the validation in human preclinical models and, most remarkably, (e) on the stringent assessment of the association of the molecular alterations with specific patient cohorts.

## Methods

### Mouse strains.

The experimental breeders used in this study were B6.129-*Ptch1^tm1Mps^* (stock no. 003081) and B6.129S2-*Trp53^tm1tyj^* (stock no. 002101) mice (The Jackson Laboratory). All mice were maintained on a C57BL/6 background for at least 5 generations prior to initiate experiments. *Ptch1*^+/–^ *p53*^+/+^ (HW), *Ptch1*^+/–^ *p53*^+/–^ (HH), and *Ptch1*^+/–^ *p53*^–/–^ (HN) mouse littermates were generated by crossing HH mice with WT/ heterozygous (WH) or with WT/null (WN) mice.

CD1-*Foxn1^nu^* and NOD.Cg-Prkdc^SCID^ (NSG) immunocompromised mice were purchased from Charles River Laboratories.

### Immunostaining on paraffin-embedded sections.

From formalin-fixed paraffin-embedded mouse and human tumor samples (University of Brescia), 2 μm sections were cut, dewaxed, and rehydrated, and endogenous peroxidase activity was blocked by 0.3% H_2_O_2_/methanol for 20 minutes. Heat-induced antigen retrieval was performed using a microwave oven or a thermostatic bath in 1.0 mM EDTA (pH 8.0), 1.0 mM citrate (pH 6.0), or 1mM Tris-EDTA (pH 9.0) buffer (all from Sigma-Aldrich; see Supplemental Methods). Sections were then washed in TBS (pH 7.4) and incubated for 1 hour or overnight in TBS/1% BSA with the specific primary antibody (see Supplemental Methods). Single immunostaining was revealed by Envision^+^System-HRP Labelled Polymer Anti-Rabbit or Anti-Mouse (DAKO) or the NovoLink Polymer Detection System (Novocastra Laboratories Ltd.), followed by diaminobenzydine (DAB) as chromogen and hematoxylin as counterstaining. For double immunostainings, after completing the first immune reaction, the second primary antibody was applied and labeled using MACH 4 Universal AP Polymer Kit (Biocare Medical); chromogen reaction was developed with Ferangi Blue Chromogen System (Biocare Medical), and nuclei were counterstained with hematoxylin.

From Carnoy-fixed human tumor samples (Istituto Neurologico Besta, Milan, Italy), 2 μm–thick sections were cut, deparaffinized, rehydrated, treated for antigen retrieval (pH 9 buffer at 98°C for 20 minutes), and incubated with normal goat serum (Agilent) and then with the primary antibody. Sections were subsequently incubated with Envision FLEX HRP-conjugated secondary antibody (Agilent), reacted with diaminobenzidine (DAB Substrate Chromogen System, Agilent), and counterstained with haematoxylin.

### Image data acquisition and analysis.

All histological and IHC staining results were reviewed independently by 2 pathologists who were blinded to the identity of the mouse samples and to the clinicopathological information of human samples. Images were acquired at 40× magnification for standard IHC and at 60× magnification for double IHC staining by a Nikon camera mounted on Nikon microscope using the NIS-Elements software. Image analysis was performed by using the open-source image processing package ImageJ-Fiji (www.fiji.sc).

### Western blotting.

Each frozen tissue/cell pellet was homogenized in 10× volume of RIPA lysis buffer (10 mM Tris-Cl [pH 7.2], 150 mM NaCl, 1 mM EDTA [pH 8]) with 1% Triton X-10/0.1% deoxycholate, 0.1% SDS (all from Sigma-Aldrich), and protease and phosphatase inhibitor mixture (Roche). Samples were then diluted in Laemmli’s SDS sample buffer (Bio-Rad). Proteins were separated by electrophoresis on 10% polyacrylamide gels according to the TGX Stain-Free FastCast Acrylamide kit protocol (Bio-Rad), and they were transferred onto Trans-Blot nitrocellulose membranes (Bio-Rad) according to the Trans-Blot Turbo Transfer System kit protocol (Bio-Rad). Primary antibodies were diluted in 3% BSA (Sigma-Aldrich) or 5% nonfat dry milk in TBS-T, and the membranes were incubated overnight at 4°C (see Supplemental Methods). The primary antibody was removed, and the blots were washed in TBS-T and then incubated for 1 hour in HRP-conjugated secondary antibodies (Amersham). Reactive proteins were visualized using a Clarity Western ECL substrate kit (Bio-Rad), and exposure was performed using UVItec (Cambridge MINI HD). Images were acquired by NineAlliance software.

### RNA-Seq.

Total RNA from mouse MB was extracted using the RNeasy Mini Kit (Qiagen) according to the manufacturer’s protocol. cDNA was synthesized starting from total RNA by QuantSeq 3′ mRNA-Seq Library Prep Kits (Lexogen). After barcoding, the RNA libraries were pooled, denatured, and diluted to 2.4 pM final concentration. RNA-Seq was performed using NextSeq 550 (Illumina) set for 76 cycles in single end (SE), yielding an average of 15 × 10^6^ clusters for each sample. Sequences were aligned using STAR (version 2.5.3a) on the reference genome GRCm38; association between reads and genes was performed by featureCounts, using GENCODE (version M13) basic annotation as reference. Normalization and analysis of count data were performed using the R-package DESeq2 (version 1.0.19) (DGE analysis based on the negative binomial distribution of counts data). The independent filtering of genes with low counts was set to a mean of 9 raw counts between all samples. The cutoff imposed for DGE was the one suggested by the FDA-led Sequencing Quality Control Consortium (SEQC) (https://www.fda.gov/science-research/bioinformatics-tools/microarraysequencing-quality-control-maqcseqc#MAQC-IIIalsoknownasSEQC), which defines a gene as differentially expressed when it has an associated FDR value lower than 0.1 (*P*_adj_ < 0.1, Benjamini-Hochberg correction) and, at the same time, the absolute value of its log_2_ fold change (FC) is greater than 1 (log_2_ FC > 1 or log_2_ FC < –1). RNA-Seq data are available at NCBI GEO (GSE183901).

### Bioinformatics analysis.

GSEAs was conducted with the JAVA Web Start GSEA platform (v.4.0.3, https://www.gsea-msigdb.org/gsea/index.jsp) and using the GSEA preranked module. Specifically, gene lists were obtained by performing DGE with DESeq2. Genes for which either FDR or log_2_ FC statistics were not available due to outliers or low counts were discarded, and the remaining genes were preranked according to the log_2_ FC. Statistics were calculated using gene set permutations (1 × 10^3^), and the classic scoring scheme was utilized. To directly test whether transcriptional differences between HW and HN MB correlated with molecular MB subgroups and subtypes, unbiased GSEA screens were performed against a total of 8 databases of gene sets (.gmx files), 4 of which directly derived from ref. 5 (i.e., SHH, WNT, Group 3, and Group 4 subgroup signatures), and the remaining were established by extracting SHH subtype–specific signatures (i.e., SHHα, SHHβ, SHHγ, and SHHδ) from the data set provided in ref. 5 (GSE85217). The online tool GEO2R was used to perform DGE analysis between each SHH subtype versus the other 3 SHH subtypes. By R Studio (v.1.2.1335) and R (v.3.5.2), only genes with *P* < 0.001 and upregulated in the considered subtype were selected for the signature. The log_2_ FC threshold was chosen to identify at least 20 genes for signature and ranged between 1 and 2. Each of the signature data sets was converted into murine orthologue genes by means of the online tool dbOrtho (https://biodbnet-abcc.ncifcrf.gov/db/dbOrtho.php). Genes that did not have corresponding murine orthologues were discarded. Gene sets with a nominal *P* value less than 0.05 and FDR-adjusted *q* value less than 0.25 were considered significant. Single gene expression analyses in publicly available patients’ data sets were performed by the R2 software (Genomics Analysis and Visualization Platform, http://r2.amc.nl).

### qPCR analysis.

Total RNA from mouse MB was extracted using the RNeasy Mini kit (Qiagen). In total, 1 μg of total RNA was reverse transcribed by using High-Capacity cDNA Reverse Transcription Kits (Applied Biosystems, Thermo Fisher Scientific). qPCR was performed by using GoTaq qPCR Master Mix (Promega), following manufacturer’s instructions. Mouse-specific primers were purchased from Sigma-Aldrich. β-Actin was used as housekeeping gene.

### Cell culturing.

Mouse CSCs were generated at the Neural Stem Cell Biology Unit after dissociation of single tumors from HW, HH, and HN mice and culturing under the conditions of the NeuroSphere Assay (25, 26). Human DAOY cells (provided by Gaetano Finocchiaro, Fondazione IRCCS Istituto Neurologico “C. Besta”, Milan, Italy) were grown as adherent monolayer in DMEM containing 20% FCS (Sigma-Aldrich).

### Evaluation of tumorigenicity.

For s.c. injection, 3 × 10^6^ to 5 × 10^6^ mouse CSCs/human DAOY cells were transferred in 100–150 μL of DMEM containing DNase (Sigma-Aldrich) and injected into the right flank of 45- to 60-day-old *nu/nu* and/or NSG mice. Mice were sacrificed at different time points between 4 and 12 weeks after injection, according to the cell line originally injected. For intracranial transplantation, 2 × 10^5^ CSCs were delivered into the right striatum by stereotactic injection through a 5 μL Hamilton microsyringe. The following coordinates were used: AV= 0; ML= +2.5mm; DV= –3.5mm from bregma. Animals were sacrificed 2–6 months after transplantation.

### Gene overexpression by viral transduction.

p53-null P7 cerebellar NSCs and HW, HH, and HN CSCs were infected with LV coding for *Rheb^Q64L^* (34) and/or RVs coding for *Myc^T58A^* and DNp53-GFP (17) for 16 hours. GFP-coding LVs/RVs were used as mock controls.

### MRI.

All the MRI studies described in the paper were performed on a small animal–dedicated 7T scanner (30/70 BioSpec; Bruker, Ettlingen). The animal protocol used to monitor tumor development in HW and HN mice included high-resolution T2-weighted sequences (TR/TE = 3000/12 ms, matrix = 170 × 170, voxel size = 0.11 mm^2^, section thickness = 0.75 mm). After converting Bruker images into the NIfTI format with MATLAB 2013 (MathWorks), tumor masses were manually segmented using ITK-SNAP (http://www.itksnap.org). Tumor volumes were then calculated by means of the *fslstats* tool of FSL (FMRIB software; Oxford Center for Functional MR Imaging of the Brain, Oxford, England).

### Treatment of autochthonous, CSC-derived, and DAOY-derived MB with rapamycin.

For in vivo administration, rapamycin (LC Laboratories) was dissolved in 100% ethanol, stored at –20°C, and diluted in a vehicle solution containing 5% Tween-80 and 5% PEG 400 (Merck) immediately before injection. Autochthonous mice were randomized based on age and/or MRI analysis and then injected i.p. with either 6 mg/kg of drug or vehicle for 5 days a week. S.c. tumor–bearing mice were randomized when tumors, as measured by a caliper, reached 20 mm^3^ in volume, and they were then treated as described above.

### Classification of human MB specimens.

Classification of human MB samples was performed by IHC using the WHO markers GAB1, Filamin A, β-catenin, p53, and c-Myc (51). Some samples from University of Brescia were also classified by nanoString technology.

### Statistics.

Results for continuous variables were expressed as mean ± SEM. Two-group comparisons were performed with the 2-tailed Student’s *t* test (independent samples, 95% CI). Three-group comparisons were performed by 1-way ANOVA followed by Tukey’s or Dunnett’s tests for multiple comparisons. In cases of nonnormal distribution, the nonparametric Mann-Whitney *U* test (nonequal SD and nonnormal distribution, 2 tails) was used. *P <* 0.05 was considered statistically significant. *P < 0.05; **P < 0.01; ***P < 0.005; ****P < 0.001. For GSEA, gene sets with a nominal *P* value less than 0.05 and FDR-adjusted *q* value less than 0.25 were considered significant.

### Study approval.

All animal experiments were approved by and performed in accordance with the guidelines of the IACUC. The retrospective study on human MB samples was conducted in compliance with the Declaration of Helsinki and with policies approved by the Ethics Boards of Spedali Civili di Brescia, University of Brescia, and Istituto Neurologico Besta. Specifically, for the retrospective and exclusively observational study on archival material obtained for diagnostic purposes, patient consent was not needed (Delibera del Garante n. 52 del 24/7/2008 and DL 193/2003).

## Author contributions

VC designed and performed experiments, acquired and analyzed data, prepared the figures, and wrote the manuscript. MC, IP, FP, and VP performed experiments, acquired data, and analyzed data. ALG, GMS, and ISP analyzed molecular data. MZ, SM, and MP performed experiments. BP, AF, AZ, AC, RM, and PLP analyzed histological, molecular, and imaging data. RG conceived and supervised the project, designed experiments, and wrote the manuscript.

## Supplementary Material

Supplemental data

## Version 1 10/21/2021

In-Press Preview

## Version 2 12/08/2021

Electronic publication
