# Supplementary material for: mTORC1 promotes malignant large cell/anaplastic histology and is a targetable vulnerability in SHH-TP53 mutant medulloblastoma
Source: JCI Insight. 2021 Dec 8;6(23):e153462. doi: 10.1172/jci.insight.153462 (PMC8675203; doi:10.1172/jci.insight.153462)
Supplement: Supplemental data [file jciinsight-6-153462-s169.pdf]

Supplementary Information

Supplementary Methods

List of antibodies for IHC and WB and detailed experimental conditions.

**IHC**				
**Antibody**	**Supplier**	**Code**	**Antigen retrieval**	**Working dilution**
mouse anti-IRF8	EBioscience	V3GYWCH	TB/EDTA	1:50
mouse anti-YAP	Santa Cruz BioTechnology	sc-101199	TB/EDTA	1:50
mouse anti-Sox2	R&D Systems	MAB2018	MW/EDTA	1:100
mouse anti-βcatenin	Merck	15B8	TB/EDTA	1:1000
rabbit anti-pS6$_{S235/236}$	Cell Signaling	#4858	TB/EDTA	1:100
rabbit anti-p4EBP1$_{T37/46}$	Cell Signaling	#2855	TB/EDTA	1:500
rabbit anti-NDRG1	Abcam	ab124689	TB/EDTA	1:100
rabbit anti-pNDRG1$_{T346}$	Cell Signaling	#5482	TB/EDTA	1:100
rabbit anti-N-Myc	Novus Biologicals	23960002	MW/EDTA	1:300
rabbit anti-c-Myc	Abcam	ab32072	TB/Tris-EDTA	1:50
rabbit anti-GPNMB	LifeSpan BioSciences	LS-B11132	TB/EDTA	1:30
rabbit anti-Gli2	Genetex	GTX27195	TB/EDTA	1:750
mouse anti-p53	Cell Signaling	#2524	TB/EDTA	1:1500
**WB**				
**Antibody**	**Supplier**	**Code**	**Blocking solution**	**Working dilution**
rabbit anti-Calnexin	Genetex	GTX13504	3% BSA	1:3000
rabbit anti-Ndrg1	Cell Signaling	#9408	3% BSA	1:500
rabbit anti-pNDRG1$_{T346}$	Cell Signaling	#5482	3% BSA	1:1000
rabbit anti-S6	Cell Signaling	#2217	3% BSA	1:2000
rabbit anti-pS6$_{S235/236}$	Cell Signaling	#4858	3% BSA	1:2000
rabbit anti-p4EBP1$_{T37/46}$	Cell Signaling	#2855	3% BSA	1:1000
rabbit anti-Tsc1	Cell Signaling	#4906	3% BSA	1:1000
rabbit anti-Tsc2	Cell Signaling	#4308	3% BSA	1:1000
rabbit anti-Tbc1d7	Cell Signaling	#14949	3% BSA	1:1000
rabbit anti-c-Myc	Cell Signaling	#5605	5% MILK	1:1000
rabbit anti-Raptor	Cell Signaling	#2280	3% BSA	1:1000
rabbit anti-pRaptor$_{S792}$	Cell Signaling	#2083	3% BSA	1:1000
mouse anti-p53	Cell Signaling	#2524	3% BSA	1:800
rabbit anti-Pten	Cell Signaling	#9188	3% BSA	1:1000
rabbit anti-Grb10	Santa Cruz BioTechnology	sc-1026	3% BSA	1:500

Legend: MW: microwave oven; TB: thermostatic bath.

Supplementary Figures

Fig. S1. Autochthonous *Ptch1*$^{+/-}$ *p53*$^{+/+}$ and *Ptch1*$^{+/-}$ *p53*$^{-/-}$ mice generate tumors with significantly different time to tumor formation

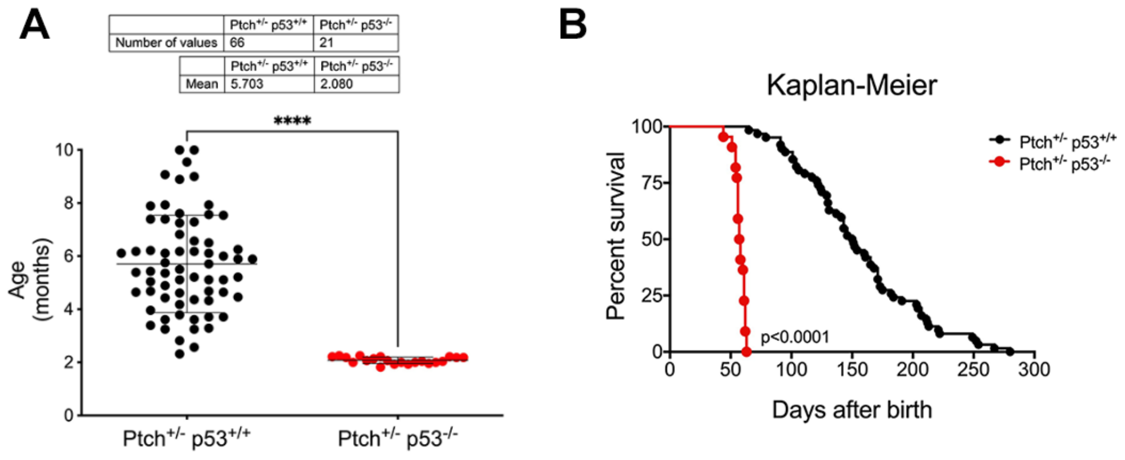

(A) Time to tumor development for the two genotypes. *Ptch1*$^{+/-}$ *p53*$^{+/+}$ mice develop MBs over a period of 2-10 months. Conversely, *Ptch1*$^{+/-}$ *p53*$^{-/-}$ mice generate tumors with short and uniform time to tumor formation (2 months after birth). Student *t* test.
(B) Kaplan-Meier survival curve for the two genotypes. Log-rank test, ***: $p<0.0001$

Related to Fig. 1

Fig. S2. Expression and activation of distinct molecular pathways in *HW* and *HN* MBs

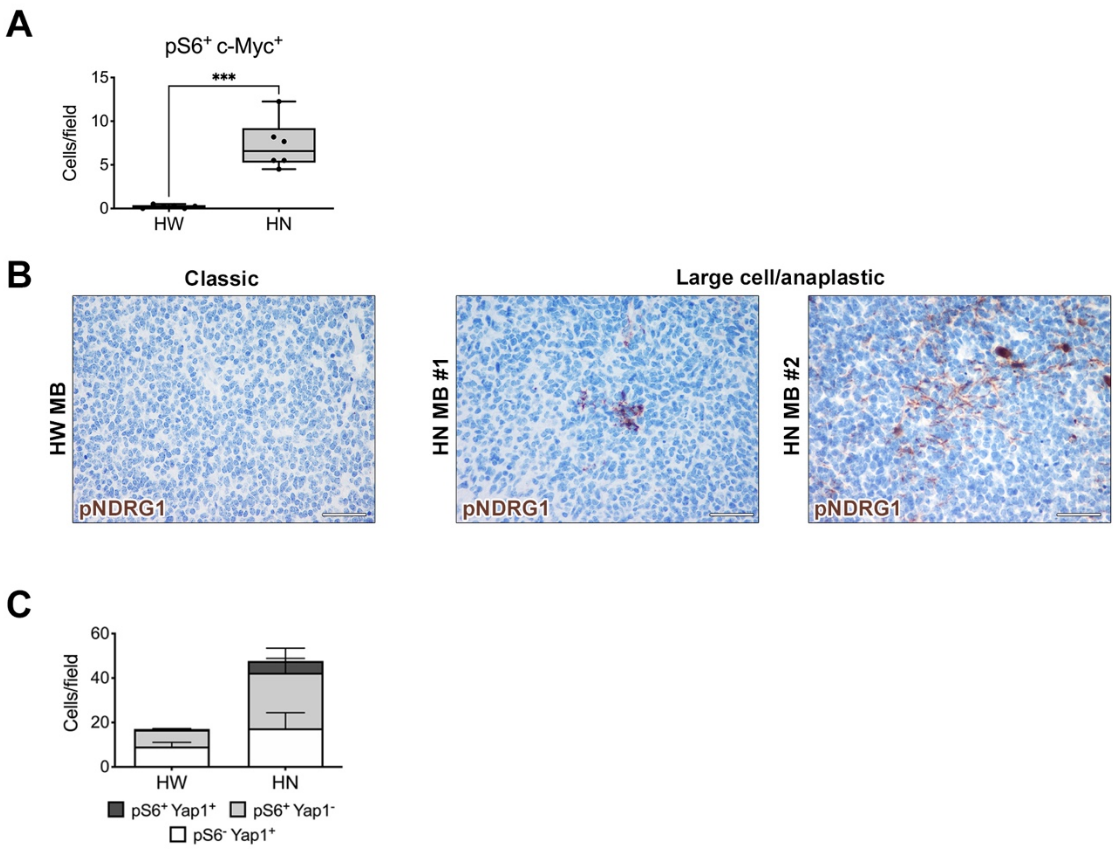

(A) Quantification of cells that are double positive for pS6 and c-Myc in *HW* and *HN* MBs. Quantitative data are represented as a box-and-whisker plot, with bounds from 25th to 75th percentile, median line, and whiskers ranging from minimum to maximum values. Student *t* test, unpaired.

(B) Focal clusters of pNdrg1 hyperactivating cells (brown) are observed in *HN* LC/A MBs but not in *HW* MBs (Scale bars: 50 µm).

(C) Quantification of cell subpopulations (shown as number of cells in a 60x microscopic field) is shown in the graphs. $pS6^+Yap1^+$ cells in *HW* vs. *HN* MBs: **=p<0.01, Student *t* test, unpaired. $pS6^+Yap1^+$ cells in *HW* vs. *HN* MBs: **=p<0.01, Student *t* test, unpaired.

Related to Fig. 1

Fig. S3. Somatic p53 mutations are detected in *HW* MBs showing LC/A histology and mTORC1 hyperactivation

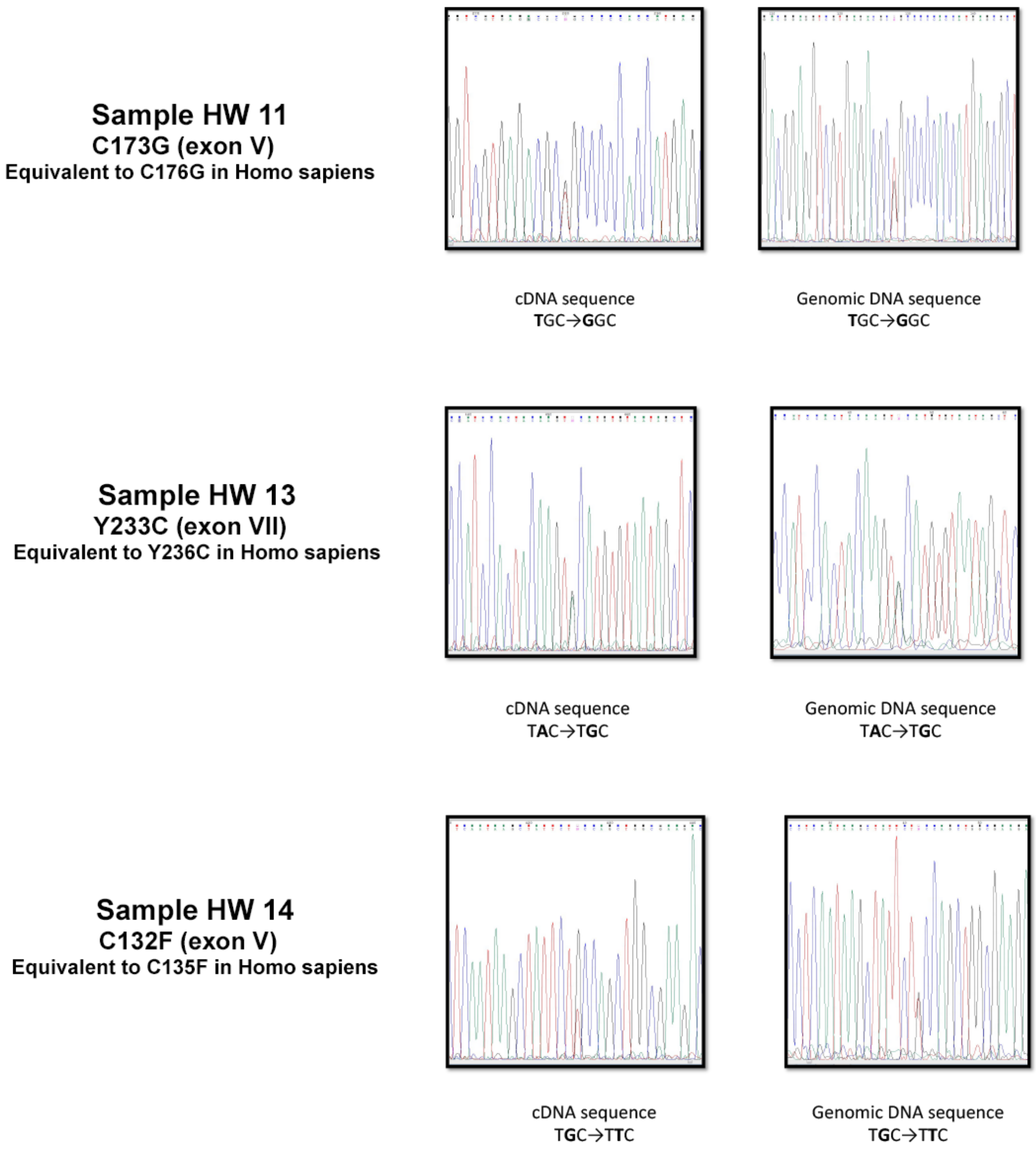

Next generation sequencing (NGS) performed on both cDNA and genomic DNA of 3 of *HW* tumor 'outliers' indicated the presence of pathogenic heterozygous somatic p53 mutations, such as C132F, Y233C and C173G.

Related to Fig. 3

Fig. S4. Hyperactivation of mTORC1 pathway in *HH* CSCs increases tumor malignancy and regulates MB subgroup specification by inducing an LC/A phenotype

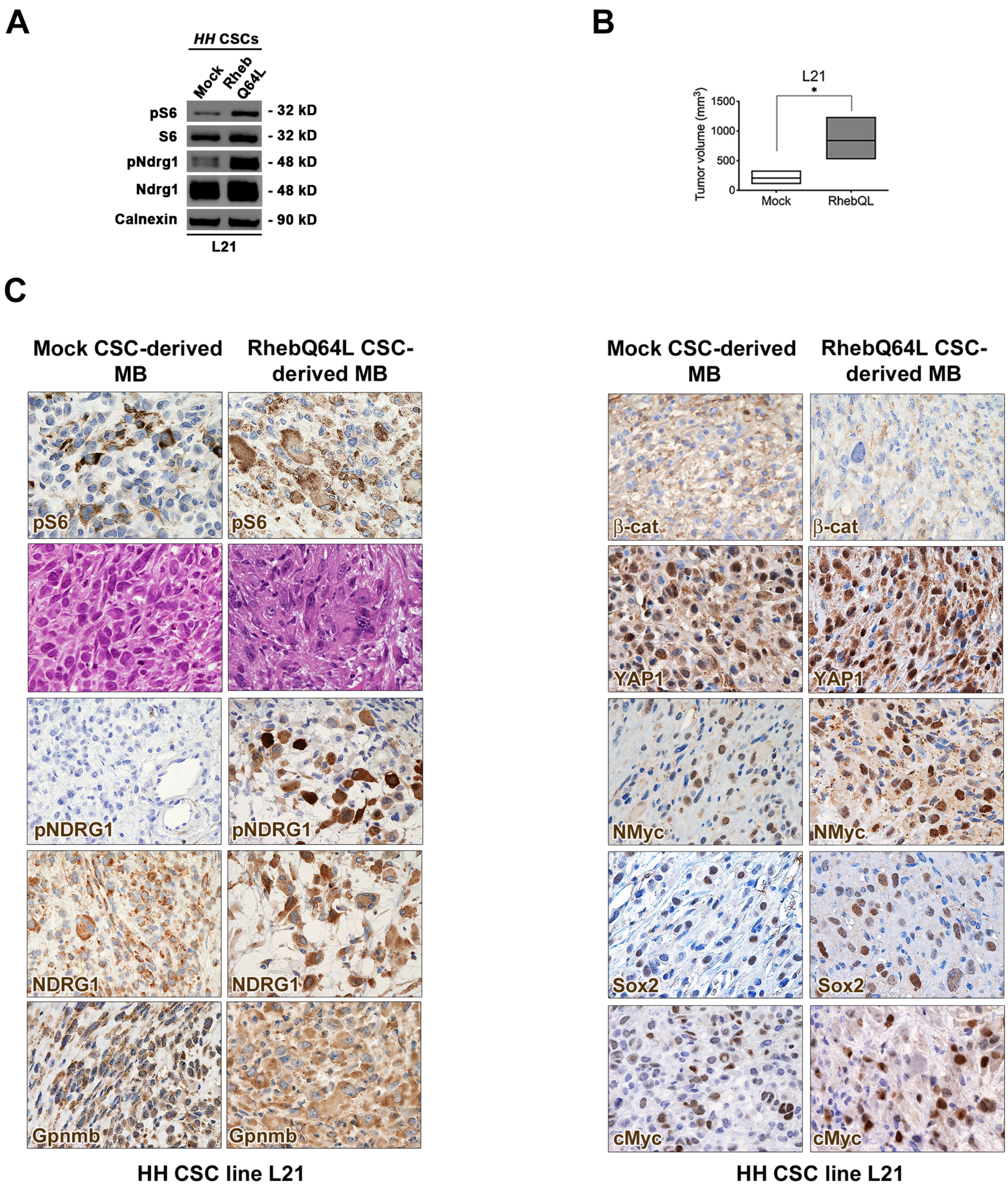

(A) WB showing that mTORC1 hyperactivation in *HH RhebQ64L* CSCs promotes also the activation of mTORC2 (CSC line L21).
(B) Hyperactivation of *Rheb*^{Q64L} in *classic HH* CSCs (L21) give rise to tumors that grow faster and larger than controls (volume measured at 54 days post-transplant for L21). Quantitative data are presented as floating bars from minimum to maximum values, line at mean. Student *t* test, unpaired.
(C) H&E staining showing pS6 hyperactivation in tumor cells only in MBs derived from *classic HH* CSCs (CSC line L21) after transduction with *RhebQ64L*. *RhebQ64L* MBs are endowed with typical *LC/A* features, *e.g.* large cells, nuclear pleomorphism, *etc.* The mTORC2 marker pNDRG1 is also strongly hyperactivated in *RhebQ64L* MBs. The mTORC1 regulated gene Gpnmb is also highly expressed in *RhebQ64L* MBs. Magnification: 600x. Left panels.
The WNT-associated classifier β-catenin is downregulated in *RhebQ64L HH* CSC-derived MBs (L21), whereas p53 mutant SHH markers as YAP1, N-Myc and Sox2 are upregulated. The Group 3 classifier cMyc is also highly expressed in *RhebQ64L HH* CSC-derived MBs. Right panels.

Related to Fig. 5

Fig. S5. Hyperactivation of mTORC1 pathway in *HN* CSCs further increases tumor malignancy and LC/A features

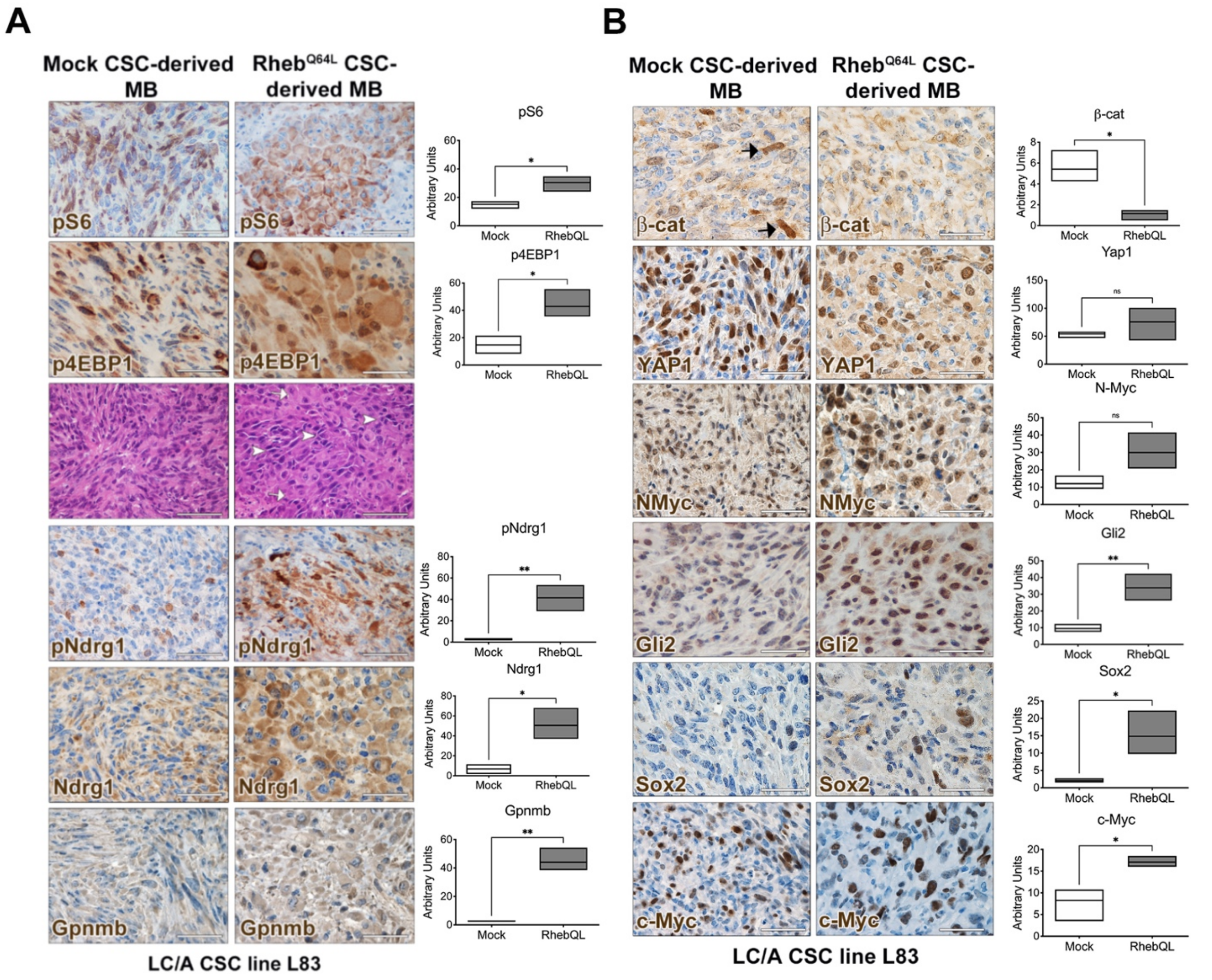

MBs derived from *LC/A HN* CSCs (L83) endogenously show high pS6 activation in tumor cells, which become more evident in *HN Rheb*^{Q64L} MBs.

(A) Additional features of *LC/A* histology, such as nuclear molding (white arrowheads), nuclear pleomorphism and enhanced frequency of large cells (white arrows), are acquired in tumors derived from *HN* CSCs after *Rheb*^{Q64L} transduction. mTORC2 activation and Gpnmb expression are also increased in the same tumors.

(B) MBs derived from *LC/A HN* CSCs (L83) after transduction with *Rheb*^{Q64L} further upregulate the expression of some subgroup-specific markers, such as Gli2, Sox2 and c-Myc.

All scale bars: 50 µm.

Quantitative data are presented as floating bars from minimum to maximum values, line at mean.

Student *t* test, unpaired.

Related to Fig. 5

Fig. S6. Hyperactivation of mTORC1 pathway in *HH* CSCs induces an LC/A phenotype

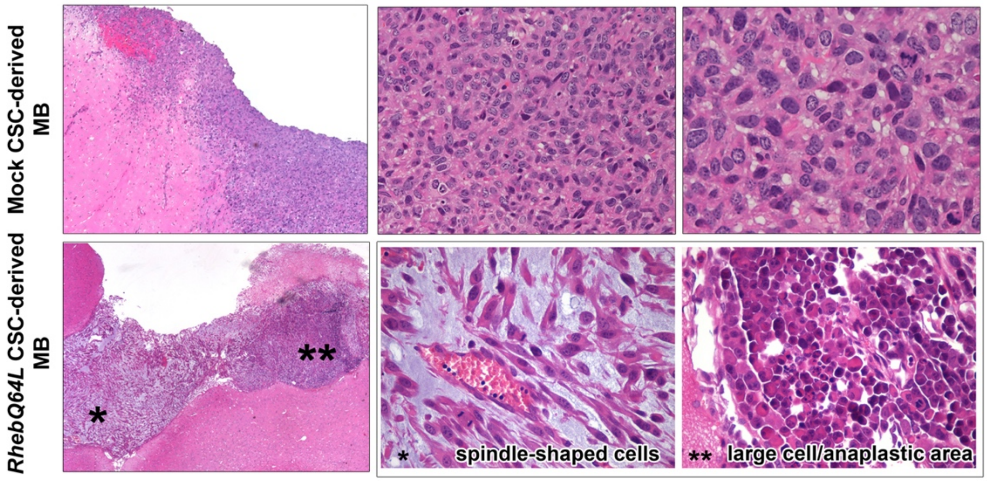

RhebQ64L-transduced CSC-derived intracranial MBs, analyzed at 53 days post-transplant, are endowed with typical LC/A features, *e.g.* nuclear molding, large cells, *etc.*, which are not retrieved in controls, analyzed at 115 days post-transplant (n=3 for each condition) (CSC line LB). Magnification: 400x, 600x.

Related to Fig. 5

Fig. S7. Pharmacological targeting of the mTOR pathway does not significantly affect the growth of autochthonous SHH p53 wildtype MBs, while is efficient in reducing tumor growth of autochthonous SHH p53 mutant MBs

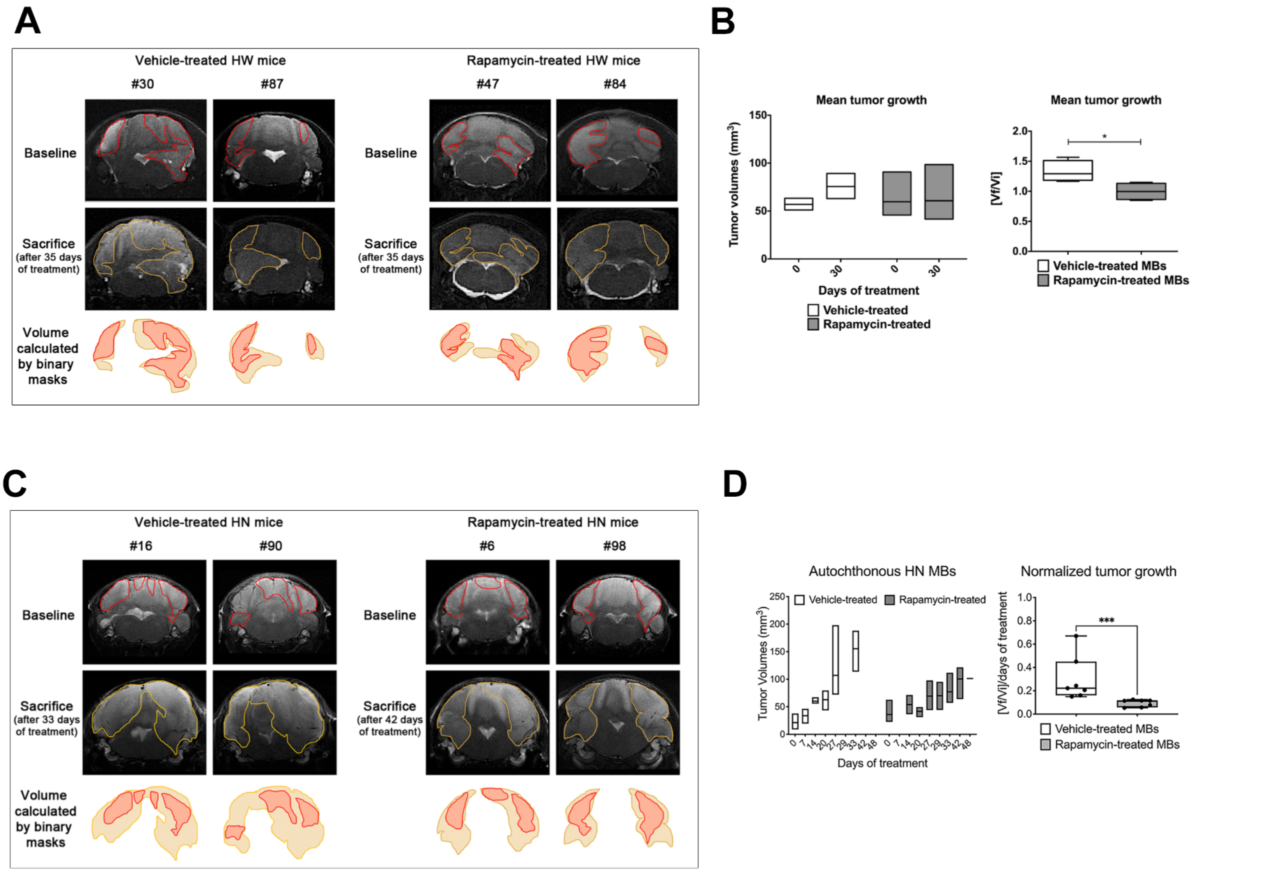

(A) Longitudinal T2-weighted MRI analysis indicates that rapamycin only slightly affects the growth of *HW* MBs. Treatment started at 4 months of age.

(B) MB development, as measured based on the volume calculated by binary masks, is not significantly affected by rapamycin treatment when compared to vehicle-treated controls, as shown by linear regression analysis of growth curves and by the incremental volume per day during rapamycin treatment. (n=5 per each group; V_f: final volume; V_i: initial volume; *: p<0.05; Mann-Whitney test).

(C) Longitudinal T2-weighted MRI analysis indicates that late rapamycin administration significantly impairs the growth of *HN* MBs. Treatment started at 25-27 days of age.

(D) MB development, based on the volume calculated by binary masks, is significantly slowed down by rapamycin treatment when compared to vehicle-treated controls, as shown by linear regression analysis of growth curves (slope values: 3.8±0.4 for controls and 1.4±0.2 for rapamycin-treated mice; F=23.496, p <0.0001) and by the incremental volume per day during rapamycin treatment (n=7 per each group; V_f: final volume; V_i: initial volume; ***: p<0.0005; Mann-Whitney test).

Related to Fig. 6

Fig. S8. Pharmacological targeting of the mTOR pathway does significantly affect the growth of autochthonous SHH p53 mutant MBs by decreasing the activation of mTORC1 and subgroup-specific markers

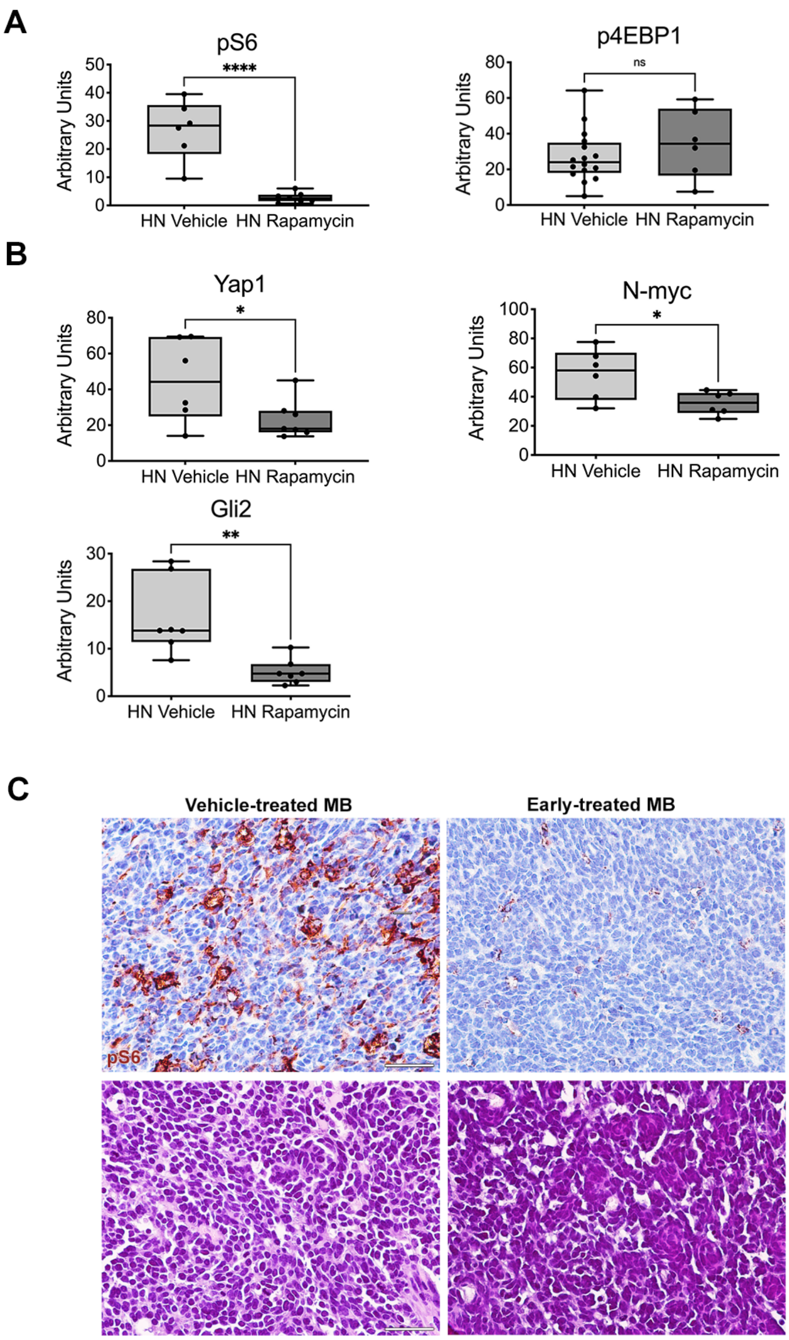

(A) Quantification of the level of pS6 and p4EBP1in *HN* MBs treated with vehicle or rapamycin is shown in the graphs.
(B) Quantification of the level of Yap1, N-Myc and Gli2 in *HN* MBs treated with vehicle or rapamycin is shown in the graphs.
(C) Following early rapamycin treatment, *HN* MBs show a significant reduction in the activation of pS6, in nuclear pleomorphism and molding. All scale bars: 50 µm.
Quantitative data are represented as a box-and-whisker plot, with bounds from 25th to 75th percentile, median line, and whiskers ranging from minimum to maximum values. Student *t* test, unpaired.

Related to Fig. 6

Fig. S9. Quantification of markers in spontaneously '*LC/A*' and enforced '*LC/A*' *Rheb*^{Q64L} *HN* and *HH* CSC-derived tumors after rapamycin treatment.

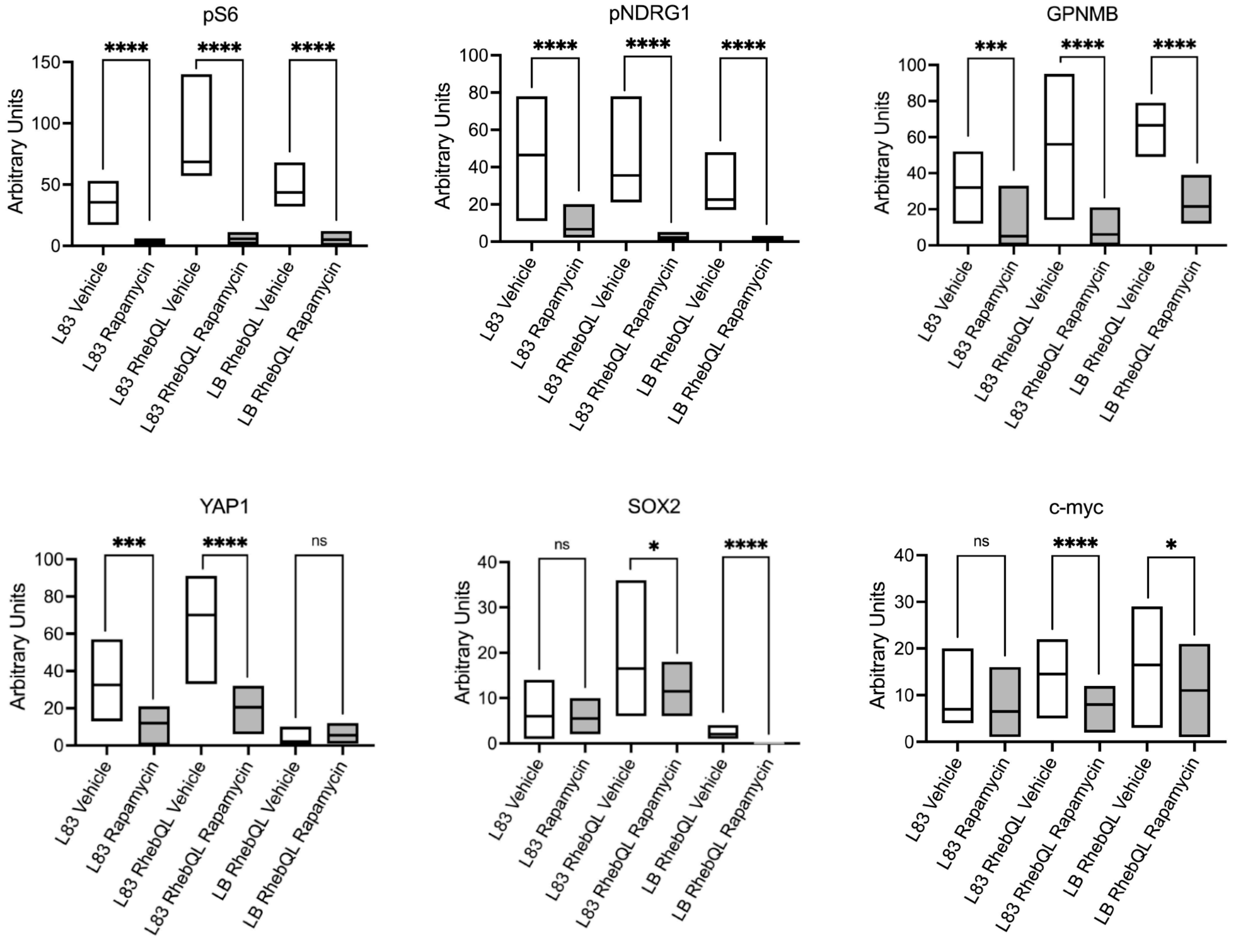

Quantification of the level of pS6, pNDRG1, GPNMB, Yap1, Sox2 and c-Myc in spontaneously '*LC/A*' and enforced '*LC/A*' *Rheb*^{Q64L} *HN* and *HH* CSC-derived tumors treated with vehicle or rapamycin is shown in the graphs. Quantitative data are presented as floating bars from minimum to maximum values, line at mean. Student *t* test, unpaired.

Fig. S10. N-MYC, GLI2 and YAP1 are significantly upregulated in SHH-*TP53* mutant MBs as compared with SHH-*TP53* wild type MBs

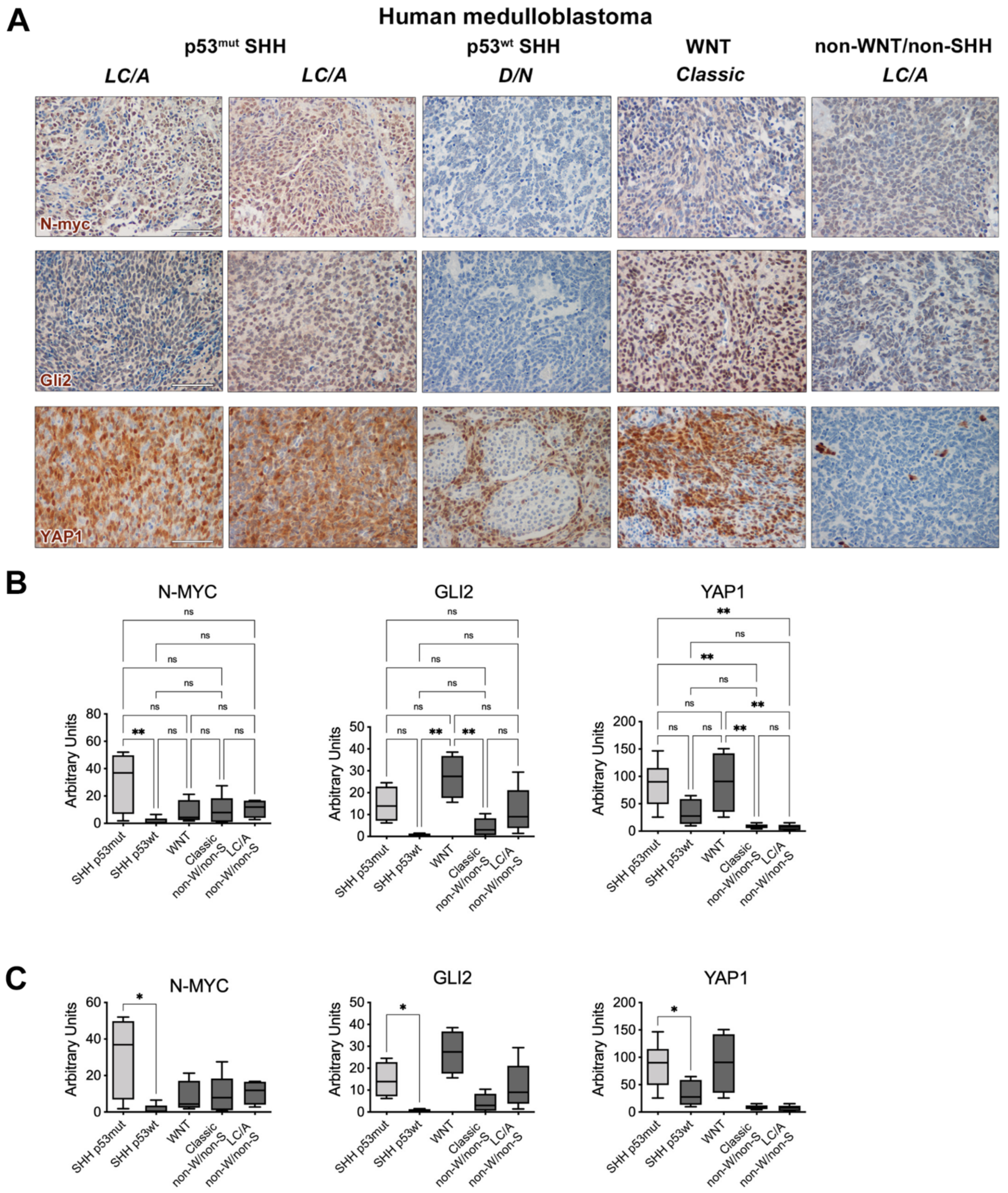

(A) N-MYC, GLI2 and YAP1 (nuclear staining, brown) are more highly expressed in human *LC/A* MBs belonging to the SHH subgroup with p53 mutation than in human desmoplastic/nodular (D/N) SHH p53wt. High expression of GLI2 and YAP1 is detected in human MBs affiliated with the WNT subgroup. Scale bars: 50 μm.
(B) Quantification of N-MYC, GLI2 and YAP1 expression in the different subgroups. Quantitative data are represented as a box-and-whisker plot, with bounds from 25th to 75th percentile, median line, and whiskers ranging from minimum to maximum values. One-way ANOVA followed by Tukey's multiple comparison test.
(C) Student *t* test, unpaired.

Related to Fig. 8

Fig. S11. Rapamycin treatment significantly impairs the growth of human LC/A MBs induced by RhebQ64L-transduced human DAOY cells

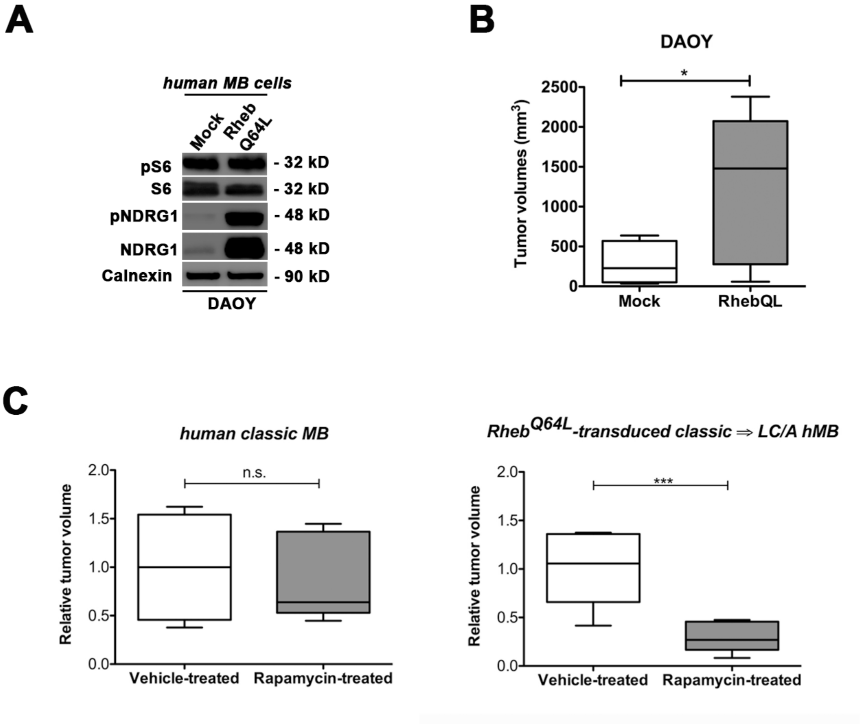

(A) WB showing that mTORC1 hyperactivation in *RhebQ64L*-transduced SHH p53 mutant DAOY cells also promotes the activation of mTORC2.
(B) Hyperactivation of *RhebQ64L* in human DAOY cells give rise to tumors that tend to grow larger than GFP-transduced controls (volume measured at 45 days post-transplant).
(C) Tumor growth is not significantly affected by rapamycin treatment in *classic* tumors generated by SHH p53 mutant human MB cells (GFP-transduced DAOY, 25 days of treatment, *n*=5), whereas it is significantly reduced in *LC/A* tumors generated by the same cells transduced with $Rheb^{Q64L}$ (DAOY, 20 days of treatment, *n*=7). Quantitative data are presented as floating bars from minimum to maximum values, line at mean. Student *t* test, unpaired.

Related to Fig. 8

Table S1. Summary of morpho-histological traits and marker-positive cells in autochthonous *HW* and *HN* mouse (m)MBs and in subgroup-specific CSC- and DAOY-derived mouse and human (h)MBs

A

	HW **mMBs**	***HN*** **mMBs**	***HH*** **CSC-derived GFP mMBs**	***HH*** **CSC-derived Rheb^{Q64L} mMBs**	***HN*** **CSC-derived GFP mMBs**	***HN*** **CSC-derived Rheb^{Q64L} mMBs**	**DAOY-derived GFP hMBs**	**DAOY-derived Rheb^{Q64L} hMBs**
**Morphological and histological features**								
**Desmoplastic**: small cells with hyperchromatic nuclei	Present	Absent	Absent	Absent	Absent	Absent	Absent	Absent
**Desmoplastic**: mature cells with abundant cytoplasm and dense intercellular neuropil	Present	Absent	Absent	Absent	Absent	Absent	Absent	Absent
**Classic**: densely packed, small "*blue*" round cells with high nuclear-to-cytoplasmic ratio	Present	Absent	Present	Absent	Absent	Absent	Present	Absent
**Large cell/ anaplastic (LC/A)**: large cells	Absent	Present	Absent	Present	Present	Present	Absent	Present
**LC/A**: enlarged angular and tightly packed cells	Absent	Present	Absent	Present	Present	Present	Absent	Present
**LC/A**: cell wrapping, nuclear molding and atypia	Absent	Present	Absent	Present	Absent	Present	Absent	Present
**LC/A**: high mitotic index and high apoptotic events	Absent	Present	Absent	Present	Present	Present	Absent	Present

B

	HW mMBs (n=52)	HN mMBs (n=21)	HW p53 mut mMBs (n=5)
**Desmoplastic/classic histology**	52/52 (100%)	0/21 (0%)	0/5 (0%)
**LC/A histology**	0/52 (0%)	21/21 (100%)	5/5 (100%)
**pS6 staining in tumor cells (> 20 IR-cells/60x field)**	0/52 (0%)	19/21(90%)	5/5 (100%)
**p4EBP1 staining in tumor cells (> 20 IR-cells/60x field)**	0/52 (0%)	19/21(90%)	5/5 (100%)

Table S2. **Gene set enrichment analysis (GSEA) report for the four distinct gene sets generated through the comparison among each MB subgroup versus the others, when tested on ranked lists of *HN vs. HW* MB genes**

NAME	SIZE	ES	NES	NOM p-val	FDR q-val	FWER p-val	RANK AT MAX	LEADING EDGE
SHH_MB_MOUSE	171	***0.292297***	***0.96***	0.5727848	0.57266665	0.953	3609	tags=23%, list=24%, signal=30%
GROUP4_MB_MOUSE	176	-0.311548	-1.38	0.0	0.0	0.0	2496	tags=30%, list=16%, signal=35%
WNT_MB_MOUSE	165	-0.296517	-1.28	0.025	0.013081396	0.002	2546	tags=24%, list=17%, signal=28%
GROUP3_MB_MOUSE	173	-0.210382	-0.96	0.54	0.5843023	0.105	1879	tags=15%, list=12%, signal=17%

A positive NES was observed for the SHH MB gene signature that was enriched in genes upregulated in *HN* MBs as compared to those in *HW* MBs.

Related to Fig. 2

Table S3. **Gene set enrichment analysis (GSEA) report for the four distinct gene sets generated through the comparison among each SHH MB subtype versus the others, when tested on ranked lists of *HN vs. HW* MB genes**

NAME	SIZE	ES	NES	NOM p-val	FDR q-val	FWER p-val	RANK AT MAX	LEADING EDGE
SHHALPHA_SIGNATURE_VS_SHH_LOGFC1_MOUSE	13	0.6709652	1.52	***0.03907380***	0.14539365	0.098	1353	tags=31%, list=9%, signal=34%
SHHBETA_SIGNATURE_VS_SHH_LOGFC2_MOUSE	16	0.5828002	1.34	0.11203319	0.22227828	0.293	768	tags=19%, list=5%, signal=20%
SHHGAMMA_SIGNATURE_VS_SHH_LOGFC2_MOUSE	50	0.38845864	1.12	0.2992611	0.41954222	0.66	3824	tags=44%, list=25%, signal=58%
SHHDELTA_SIGNATURE_VS_SHH_LOGFC2_MOUSE	17	0.43500158	1.02	0.44808742	0.43961504	0.789	2486	tags=35%, list=16%, signal=42%

A significant NOM p-value (< 0.05) was retrieved for the SHH alpha gene dataset (highlighted in bold italic).

Related to Fig. 2

Table S4. Gene set enrichment analysis (GSEA) report for the two distinct gene sets generated through the comparison among SHHα p53 mutant LC/A MBs versus SHHα p53 wild-type classic/desmosplastic MBs, when tested on ranked lists of *HN vs. HW* MB genes

NAME	SIZE	ES	NES	NOM p-val	FDR q-val	FWER p-val	RANK AT MAX	LEADING EDGE
SHHA_LCA_P53MUT_VS_DESMOCL_P53WT_LOGFC1.5_MOUSE	15	0.57	1.33	0.121	***0.121***	0.086	768	tags=20%, list=5%, signal=21%

A significant FDR q-value (< 0.25) was retrieved for the SHH alpha p53 mutant LC/A MB gene dataset (highlighted in bold italic).

Related to Fig. 2

Table S5 – Statistical analysis of marker quantification for *HH, HN* and *Myc* CSC-derived tumors.

Pairwise comparison	P value					
	pS6		c-myc		$pS6^+$ $IRF8^-$	
LB vs. L84	0.8249	ns	0.0002	***	0.3295	ns
LB vs. ML9	0.9813	ns	0.0001	****	0.9995	ns
LB vs. L83	0.0002	***	0.9319	ns	0.0001	****
LB vs. L66	0.0006	***	0.0619	ns	0.0001	****
L84 vs. ML9	0.9849	ns	0.5482	ns	0.5468	ns
L84 vs. L83	0.0024	**	0.0009	***	0.0001	****
L84 vs. L66	0.0052	**	0.1513	ns	0.0001	****
ML9 vs. L83	0.0008	***	0.0001	****	0.0001	****
ML9 vs. L66	0.0018	**	0.0079	**	0.0001	****
L83 vs. L66	0.9999	ns	0.2366	ns	0.0625	ns

One-way ANOVA followed by Tukey's multiple comparison test.

Related to Fig. 4

Table S6. Demographic, histological and molecular information of MB patients

	ID	Sex	Age at diagnosis	Classification by IHC	Classification by nanoString	Diagnosis	Staining for pS6
1	2059.1/00 A.3	F	8	**SHH p53 mut**	SHH	Desmoplastic with many LC/A areas	Positive
2	6024/02 K.1, 6027/02 A.1	M	8	**SHH p53 mut**		LC/A	Positive
3	16091.2/07 A.1	M	20	**SHH p53 mut**		LC/A	Positive
4	12352/98 BS	F	3	**SHH p53 mut**	SHH	Desmoplastic with many LC/A areas	Positive
5	2709/2009 A5	M	3	**SHH p53 mut**		Desmoplastic with many LC/A areas	Positive
6	2743/2016	M	10	**SHH p53 mut**		LC/A	Positive
7	39240 Besta	F	5	**SHH p53 mut**		LC/A	Positive
8	40161 Besta	M	24	**SHH p53 mut**		LC/A	Positive
9	8151/00 A.3	M	1	**SHH p53 wt**		Desmoplastic/ Nodular	Negative
10	8665/00 A.2	F	2	**SHH p53 wt**		Desmoplastic/ Nodular	Negative (positive in stroma)
11	15189/01 A.6	M	5	**SHH p53 wt**		Desmoplastic/ Nodular	Negative
12	719/99 BS	F	5	**SHH p53 wt**	SHH	Desmoplastic/ Nodular	Negative
13	502/2010	F	2	**SHH p53 wt**	SHH	Desmoplastic/ Nodular	Negative
14	1847/2011	M	2	**SHH p53 wt**		Desmoplastic/ Nodular	Negative
15	9242/12 A.16	M	9 months	**SHH p53 wt**		Desmoplastic/ Nodular	Negative
16	22510/16	M	2	**SHH p53 wt**		Desmoplastic/ Nodular	Negative
17	1386/2017	M	3	**SHH p53 wt**		Desmoplastic/ Nodular	Negative (positive in stroma)
18	10940/97 BS	M	3	**SHH p53 wt**	SHH	Desmoplastic/ Nodular	Negative
19	1623/2007	F	1	**SHH p53 wt**		Desmoplastic/ Nodular	Negative
20	3766/2013	M	2	**SHH p53 wt**		Desmoplastic/ Nodular	Negative
21	8196.1/02 A.3	F	2	**SHH p53 wt**	SHH	Classic	Negative (positive in vessels)
22	10140.2/09 A.5	F	31	**SHH p53 wt**		Classic	Negative (positive in stroma)
23	1072/99 BS	M	12	**SHH p53 wt**	SHH	Desmoplastic with focal anaplasia	Positive in focal areas

24	14884/01 A.1	M	5	**WNT**		Classic	Negative (positive in stroma)
25	12856.1/03 K.1, 12859/03 A.3	F	7	**WNT**		Classic	Negative
26	4808.3/06 A.2	M	4	**WNT**		Classic	Negative (positive in stroma)
27	10557/01 BS	M	9	**WNT**	WNT	Classic	Negative
28	42041 Besta	M	34	**Non WNT/ non SHH**		Classic	Negative
29	12530/99 K.1	M	8	**Non WNT/ non SHH**	Gr3	Classic	Negative (positive in stroma)
30	10225.2/00 A.2	M	7	**Non WNT/ non SHH**		Classic	Negative
31	8850.1/01 A.2; 8930/01 A.1	M	13	**Non WNT/ non SHH**		Classic	Negative (positive in stroma)
32	14754/01 A.4	M	2	**Non WNT/ non SHH**		Classic	Negative
33	6421.1/02 A.1	M	2	**Non WNT/ non SHH**		Classic	Negative
34	6442/02 A.1	F	19	**Non WNT/ non SHH**		Classic	Negative (positive in stroma)
35	7844/02 B.1	M	10	**Non WNT/ non SHH**	Gr3	Classic	Negative
36	8657.1/02 A.2; 1927/02	M	9	**Non WNT/ non SHH**	Gr4	Classic	Negative (positive in stroma)
37	11627/02 A.1; 2582/02	M	7	**Non WNT/ non SHH**	Gr4	Classic	Negative
38	12330/02 A.3	M	7	**Non WNT/non SHH**		Classic	Negative (positive in stroma)
39	2468/06 A.1	M	5	**Non WNT/ non SHH**		Classic	Negative
40	15553.3/06 A.1	M	32	**NON (p53 mutant clone and p53wt clone)**		Classic	Negative
41	7775.3/09 A.2	M	7	**Non WNT/ non SHH**		Classic	Negative (positive in stroma)
42	93/2003	M	11	**Non WNT/ non SHH**		Classic	Negative
43	2768/2003	M	9	**Non WNT/ non SHH**	Gr4	Classic	Negative
44	2913/2004	M	6	**Non WNT/ non SHH**	Gr4	Classic	Negative
45	437/2006	M	5	**Non WNT/ non SHH**		Classic	Negative
46	2262/2006 A14	M	9	**Non WNT/ non SHH**		Classic	Negative

47	4193/2006	M	23	**Non WNT/ non SHH**		Classic	Negative
48	1255/2007 A18	F	7	**Non WNT/ non SHH**		Classic	Negative
49	2300/2007	M	8	**Non WNT/ non SHH**		Classic	Negative
50	4023/2007	F	3	**Non WNT/ non SHH**	Gr3	Classic	Negative
51	1473/2008	F	10	**Non WNT/ non SHH**	Gr4	Classic	Negative
52	1207/2009 A4	F	3	**Non WNT/ non SHH**	Gr4	Classic	Negative
53	3350/2009 A7	M	2	**Non WNT/ non SHH**	Gr3	Classic	Negative
54	3609/2009	F	14	**Non WNT/ non SHH**	Gr4	Classic	Negative
55	29/2010	F	5	**Non WNT/ non SHH**	Gr3	Classic	Negative
56	562/2010	F	7	**Non WNT/ non SHH**		Classic	Negative
57	3287/2011 (P)	M	9	**Non WNT/ non SHH**	Gr4	Classic	Negative
58	2200/2012 (R)	M	10	**Non WNT/ non SHH**		Classic	Negative
59	2069/2012	M	11	**Non WNT/ non SHH**	Gr4	Classic	Negative
60	3975/2014	F	4	**Non WNT/ non SHH**		Classic	Negative
61	1812/2015	F	6	**Non WNT/ non SHH**		Classic	Negative
62	3025/2016	F	3	**Non WNT/ non SHH**		Classic	Negative
63	1924/2017 A3	M	6	**Non WNT/ non SHH**		Classic	Negative
64	3177/2017	M	4	**Non WNT/ non SHH**		Classic	Negative (positive in stroma)
65	2518/2003	F	10	**Non WNT/ non SHH**		Classic with mature areas	Negative
66	3181/2003	M	13	**Non WNT/ non SHH**		Classic with mature areas	Negative
67	2/2010	F	6	**Non WNT/ non SHH**	Gr3	Classic with neuronal differentiation	Negative
68	125/2010	M	6	**Non WNT/ non SHH**	Gr3	Classic with neuronal differentiation	Negative
69	6644/96 BS	M	13	**Non WNT/ non SHH**	Gr4	Classic with neuronal differentiation	Negative
70	1622/99 BS	M	5	**Non WNT/ non SHH**	Gr4	Classic with neuronal and glial differentiation	Negative (positive in stroma)
71	22483.2/09 A.1	M	6	**Non WNT/ non SHH**		Classic with focal desmoplastic areas	Negative

72	41654/13 A.1	M	6	**Non WNT/ non SHH**		Classic with focal desmoplastic areas and LC/A	Negative (positive in stroma)
73	1852/2003	M	8	**Non WNT/ non SHH**		Classic with LC/A areas	Negative
74	16312.2/14 K.1	M	53	**Non WNT/ non SHH**		Classic with LC/A areas	Negative
75	21598.1/14 K.1	M	5	**Non WNT/ non SHH**		Classic with LC/A areas	Negative (positive in stroma)
76	17803.1/06 A.1	M	12	**Non WNT/ non SHH**		Desmoplastic/Nodular	Negative
77	5328.2/16 A.1	F	5	**Non WNT/ non SHH**		Desmoplastic/Nodular	Negative
78	12481.2/07 K.1	M	14	**Non WNT/ non SHH**		MBEN	Negative
79	12913/02 A.1	M	3	**Non WNT/ non SHH**		LC/A	Negative (positive in stroma)
80	7039.2/02 A.2 (P) 15818/03 A5 (R)	F	7	**Non WNT/ non SHH**		LC/A	Positive in focal areas
81	12666/97 BS	M	3	**Non WNT/ non SHH**	Gr3	LC/A	Negative
82	9715/98 BS	F	5	**Non WNT/ non SHH**	Gr4	LC/A	Negative (positive in stroma)
83	251/2004	M	4	**Non WNT/ non SHH**	Gr4	LC/A with rhabdoid differentiation	Negative
84	3394/2009	F	4	**Non WNT/ non SHH**	Gr3	LC/A	Negative
85	1297/2010	M	15	**Non WNT/ non SHH**	Gr4	LC/A	Negative (positive in stroma)
86	3011/2011	M	5	**Non WNT/ non SHH**	Gr3	LC/A	Negative (positive in stroma)
87	2690/2012	M	2	**Non WNT/ non SHH**		LC/A	Negative
88	3579/2012	M	12	**Non WNT/ non SHH**	Gr4	LC/A	Negative
89	359/2014	M	4	**Non WNT/ non SHH**		LC/A	Negative
90	3137/2017	M	4	**Non WNT/ non SHH**		LC/A	Negative

Legend: (P): primary tumor; (R): recurrence

Related to Fig. 8

Table S7 - Gene set enrichment analysis (GSEA) report for two distinct gene sets related to mTOR, when tested on ranked lists of genes differentially expressed between human SHHα MBs with LC/A histology *vs* human SHHα MBs with desmoplastic/classic histology.

NAME	SIZE	ES	NES	NOM p-val	FDR q-val	FWER p-val	RANK AT MAX	LEADING EDGE
REACTOME_MTORC1_ MEDIATED_SIGNALLING	22	0.59	1.63	0.010	0.010	0.005	3907	tags=45%, list=18%, signal=55%
REACTOME_MTOR_ SIGNALLING	38	0.50	1.56	0.018	0.018	0.009	3907	tags=39%, list=18%, signal=48%

A significant FDR q-value (< 0.25) was retrieved for both mTOR-related datasets.

Related to Fig. 8

List S1A. Genes upregulated in *HW* tumors *vs.* *HN* tumors

GeneID	log2FoldChange	pvalue	padj	mark_SEQC
**Xist**	8,802648363	1,48403E-07	7,21041E-05	1
**2010300F17Rik**	6,710091873	1,4505E-09	1,76187E-06	1
**Lcn2**	5,796845519	6,86734E-11	1,43036E-07	1
**Foxd1**	4,492413861	2,90257E-07	0,000120879	1
**Mmp3**	4,00627185	0,001848432	0,082393708	1
**Tgm1**	3,351326246	2,27619E-05	0,003529545	1
**Prdm13**	3,322384988	0,000400531	0,029045454	1
**Cd5l**	3,287471501	0,000396557	0,029045454	1
**Cxcl5**	3,281750069	3,87794E-05	0,005117282	1
**Steap4**	3,001205581	5,79317E-06	0,001361103	1
**Gdpd3**	2,992125104	0,000288812	0,022625103	1
**Serpina3n**	2,8875143	7,15954E-11	1,43036E-07	1
**Tbx4**	2,792830722	0,000399078	0,029045454	1
**Atp6ap1l**	2,751133843	0,003823797	0,130933942	1
**Cxcl1**	2,70906277	0,001163191	0,062105027	1
**Gm45836**	2,680059286	5,08004E-05	0,006275137	1
**Mroh2a**	2,664893316	1,84676E-08	1,57716E-05	1
**Aoah**	2,562660321	4,17639E-05	0,005339911	1
**Cdkn1a**	2,558921332	1,06525E-06	0,000388177	1
**Cbln4**	2,509020167	7,96331E-08	4,29901E-05	1
**Xlr3a**	2,372940563	0,003764568	0,130648439	1
**Atp6v0d2**	2,372681956	1,57626E-05	0,002671579	1
**Neu4**	2,330178529	7,85047E-11	1,43036E-07	1
**Pcdhgb5**	2,239863229	0,002658592	0,105878794	1
**B430306N03Rik**	2,080981506	0,001212143	0,063784084	1
**Shtn1**	2,077713112	9,3641E-06	0,00189571	1
**Chil1**	2,059661997	0,002068757	0,089414079	1
**Pqlc3**	2,045075691	0,002812826	0,110214398	1
**Gucy2f**	2,018843774	0,001342037	0,067921991	1
**Vwa3b**	2,008208393	0,00603159	0,177609013	1
**Fam89a**	2,001675051	0,003539951	0,124583865	1
**Ms4a4a**	1,989598177	0,002762186	0,109110071	1
**Wfdc17**	1,968506839	0,006070065	0,177779907	1
**Col20a1**	1,960671238	0,00286969	0,1112463	1
**Cp**	1,883628059	4,91687E-05	0,00612549	1
**Gng4**	1,838040334	0,000686867	0,042603288	1
**BC067074**	1,816554943	0,006073984	0,177779907	1
**Pirb**	1,770589064	0,006486021	0,186102825	1
**Cela1**	1,690344654	1,52646E-05	0,002648771	1
**Frmd5**	1,689903363	1,45856E-06	0,000494418	1
**Ccdc88c**	1,682898938	0,000308734	0,023684754	1
**Hrk**	1,679663581	4,80503E-06	0,00121105	1
**Erbb3**	1,678187607	0,000922315	0,052107237	1
**Simc1**	1,663790405	3,58631E-08	2,37609E-05	1
**Plch1**	1,64201577	1,09634E-05	0,002102658	1
**Vcan**	1,6384172	1,63939E-06	0,000543085	1
**9030617O03Rik**	1,618253574	0,00018375	0,016257482	1
**Irx1**	1,613620644	0,000632989	0,040114978	1
**Cmtm8**	1,601458741	0,008450792	0,221544509	1

**Rlbp1**	1,593152542	6,00089E-07	0,000230181	1
**Myof**	1,58272478	0,004233452	0,140883086	1
**Gpr17**	1,574992973	4,83596E-06	0,00121105	1
**Cd38**	1,55280759	1,09475E-05	0,002102658	1
**Egfem1**	1,551265772	0,005795435	0,174533604	1
**Lyz2**	1,551237457	1,42019E-06	0,000492874	1
**Lhx2**	1,549464441	0,000447073	0,031110443	1
**Nxph1**	1,525000305	6,77399E-06	0,001469718	1
**Slc2a13**	1,513286377	0,000396021	0,029045454	1
**Trp53**	1,507595361	6,6445E-12	2,64895E-08	1
**Galnt3**	1,495342795	0,002201531	0,093829012	1
**Susd5**	1,488065006	6,98059E-05	0,008075319	1
**Gm26911**	1,481262328	0,00769354	0,208053863	1
**Eda2r**	1,460113539	0,000109667	0,011498303	1
**AB124611**	1,451640393	0,007408022	0,204494026	1
**C4b**	1,446845625	1,82297E-07	8,30361E-05	1
**Nol3**	1,440364181	0,000721931	0,043482917	1
**Csf2rb**	1,439674448	0,000370451	0,027409645	1
**Xdh**	1,434971149	0,001234506	0,064727187	1
**Cplx2**	1,429815121	3,63842E-06	0,001036686	1
**3110035E14Rik**	1,417068393	3,615E-05	0,004970961	1
**Col28a1**	1,411367914	0,001644569	0,078082199	1
**Tnr**	1,387617037	4,91566E-05	0,00612549	1
**Syndig1l**	1,386266512	0,00022802	0,018992127	1
**Ret**	1,368534544	0,004699648	0,151889286	1
**Phactr1**	1,327191798	1,62429E-12	2,36756E-08	1
**F2r**	1,32266908	1,12223E-05	0,002124373	1
**Fgd3**	1,315496186	5,52385E-06	0,001319929	1
**Snx22**	1,307901319	0,000177781	0,016081076	1
**Trip13**	1,284769854	8,57958E-10	1,13687E-06	1
**Iqgap2**	1,272569644	0,000190699	0,016695182	1
**Dusp14**	1,266143505	0,000299068	0,023064609	1
**C1ql1**	1,265897868	0,001259349	0,065558121	1
**Rbm24**	1,262013164	0,00810663	0,214902685	1
**Gprin1**	1,232268884	7,01128E-10	1,02196E-06	1
**Cenph**	1,222997288	0,001788988	0,080731551	1
**Itih3**	1,220399243	0,000698774	0,0427123	1
**Ptch1**	1,212499539	0,000111815	0,011498303	1
**Srd5a1**	1,194074317	1,63532E-07	7,68918E-05	1
**Arl10**	1,189361254	1,38024E-06	0,000490692	1
**Abcc3**	1,182524616	2,04508E-05	0,003275722	1
**Nop16**	1,172461373	4,16493E-08	2,63948E-05	1
**Ptpro**	1,170202164	0,003313123	0,119762894	1
**Doc2a**	1,168403684	0,001614731	0,076916076	1
**Ttc37**	1,166959821	8,37178E-05	0,009244479	1
**Tgm2**	1,162962321	0,002214414	0,094102909	1
**Golm1**	1,156313919	2,87283E-05	0,004154384	1
**Depdc1b**	1,15455319	0,008108979	0,214902685	1
**Polk**	1,146261978	0,000690405	0,042641313	1
**Cecr6**	1,134438951	0,003493539	0,12379365	1
**Cltb**	1,126464323	0,000235512	0,01931616	1
**Lhfpl3**	1,119688219	2,09953E-05	0,003326387	1
**Enc1**	1,116950909	0,000134358	0,012884201	1

**Ankrd34b**	1,115733476	0,005832358	0,175283399	1
**Il18bp**	1,111543542	0,000329823	0,024780916	1
**Nfil3**	1,110497059	0,006859726	0,193399168	1
**Sox10**	1,10319324	0,00019128	0,016695182	1
**Dhx29**	1,100910779	0,000706204	0,0427123	1
**Matn4**	1,100121872	0,001686744	0,078549466	1
**Smad5**	1,098935404	6,70189E-06	0,001469718	1
**Bcas1**	1,097958031	4,1284E-05	0,005325267	1
**Msh3**	1,096654005	2,22392E-05	0,003485581	1
**Irx2**	1,084996941	0,002773109	0,109245486	1
**Poc5**	1,082667975	0,000171758	0,015946118	1
**Camk2b**	1,082497691	0,002617695	0,105040113	1
**Hk2**	1,079451403	1,3112E-05	0,002419249	1
**Hfe**	1,078915082	0,000857467	0,049206478	1
**Frmd4b**	1,077203809	0,000457703	0,031618415	1
**Med10**	1,07613721	2,09403E-07	8,97723E-05	1
**Uimc1**	1,070785293	6,35985E-08	3,56543E-05	1
**Pdcd6**	1,069591728	1,47006E-05	0,002613113	1
**Agtpbp1**	1,069567822	2,29939E-08	1,764E-05	1
**Cd22**	1,067800045	0,009107624	0,232085175	1
**Serinc5**	1,06768318	0,000235886	0,01931616	1
**Col23a1**	1,066805959	3,72499E-05	0,005015645	1
**Ccdc127**	1,062943174	4,76131E-08	2,8917E-05	1
**Brd9**	1,055166846	9,72589E-08	5,06302E-05	1
**Pllp**	1,052627889	0,001082074	0,058415941	1
**Utp15**	1,046907682	1,90024E-06	0,000602129	1
**Pcdh18**	1,043497926	0,001664753	0,078549466	1
**4833420G17Rik**	1,042101723	0,000268327	0,021714604	1
**H2afy**	1,041353476	5,12269E-07	0,000201806	1
**Thoc3**	1,038807831	1,01375E-05	0,002024159	1
**Caml**	1,038296183	2,84537E-06	0,00084641	1
**Pak1ip1**	1,030388102	5,88292E-06	0,001361103	1
**Dbn1**	1,030185869	1,05306E-05	0,002074245	1
**Parp8**	1,023740259	0,000324543	0,024638214	1
**Matn2**	1,023246227	0,002403153	0,099558223	1
**Nsd1**	1,022049813	0,001772488	0,08023535	1
**Rab3c**	1,0174942	2,21647E-06	0,000687388	1
**Slc22a23**	1,017055833	0,002005264	0,087511172	1
**Rftn2**	1,016753217	0,001523516	0,073776628	1
**Pde4d**	1,007204144	0,002172862	0,093374212	1
**Pdzrn3**	1,006287397	0,007649475	0,207246746	1
**Zbed3**	1,006082068	1,50685E-05	0,002646239	1
**Zcchc6**	1,003392722	0,000289471	0,022625103	1
**Nln**	1,002053031	0,001589554	0,075965032	1
**Fcgr2b**	1,001289736	0,000803456	0,046472942	1
**Cacnb3**	1,000107919	0,003177879	0,118781469	1

Related to Fig. 2

List S1B. Genes downregulated in *HW* tumors *vs.* *HN* tumors

GeneID	log2FoldChange	pvalue	padj	mark_SEQC
**Lhx9**	-12,049938	2,645E-05	0,00396115	-1
**Dcpp1**	-10,639838	0,00031565	0,02408864	-1
**Dcpp2**	-10,002049	0,00067934	0,04231837	-1
**Dcpp3**	-7,5986906	0,00012286	0,01209966	-1
**Vsx2**	-6,2946463	2,6429E-10	4,2803E-07	-1
**Igf2bp3**	-6,1534328	3,9068E-07	0,00015818	-1
**Fgf8**	-4,6220975	7,5226E-05	0,00860172	-1
**Ripply1**	-4,561561	4,0644E-05	0,00528959	-1
**Robo3**	-4,3121676	7,2693E-12	2,6489E-08	-1
**Ifi44**	-4,1665341	1,3652E-05	0,00248431	-1
**Ssu2**	-4,0642134	0,00011281	0,0114983	-1
Otx1	-3,9331912	1,1986E-07	6,0244E-05	-1
**Pcdha12**	-3,5440727	5,9781E-05	0,00708432	-1
**Mir6236**	-3,5363131	0,00074563	0,04436018	-1
**Usp18**	-3,4787401	2,724E-08	1,9755E-05	-1
**Zfp977**	-3,4467075	0,00134031	0,06792199	-1
**Ifi27l2a**	-3,4117126	0,00020021	0,01715721	-1
**Clvs2**	-3,3290378	0,00034942	0,02611865	-1
**Zfp947**	-3,2366624	0,00309622	0,11719777	-1
**Scml4**	-3,1681573	0,00294765	0,11366388	-1
**Sp5**	-3,1532476	4,91E-06	0,00121105	-1
**Zfp820**	-3,1033673	0,00023306	0,01930146	-1
**Mir124-2hg**	-3,0920713	1,5423E-11	4,4961E-08	-1
**Cth**	-3,0276153	4,9718E-08	2,8988E-05	-1
**Iigp1**	-2,9334098	3,479E-05	0,00482955	-1
**Irf7**	-2,9234867	3,6691E-09	3,7457E-06	-1
**Cck**	-2,9048083	0,00661722	0,18912272	-1
**Lars2**	-2,8470822	2,891E-05	0,00415438	-1
**Pabpc4l**	-2,8321734	0,00303899	0,11596476	-1
**Zfp979**	-2,8304999	0,00848091	0,22193503	-1
Dach2	-2,8171836	0,00042564	0,02997151	-1
**Cldn2**	-2,7942715	0,00245697	0,10059756	-1
**AU041133**	-2,7537682	0,00079981	0,04647294	-1
**Cfap61**	-2,7441943	0,00331349	0,11976289	-1
**Oas1b**	-2,7210505	4,4746E-05	0,0056714	-1
**Tenm3**	-2,7017146	0,00267392	0,10591048	-1
**Zfp976**	-2,68272	0,00011147	0,0114983	-1
**Bdh2**	-2,666834	0,00509838	0,15913061	-1
**Mdk**	-2,6309659	9,1892E-06	0,00188651	-1
**Ifit1**	-2,6289442	0,00062151	0,03965913	-1
**Egr1**	-2,6144107	0,00015245	0,01433608	-1
**Rmst**	-2,5802484	0,0002859	0,0226251	-1
**Aspa**	-2,5601572	0,00389395	0,13230349	-1
**Zfp677**	-2,5564243	7,5538E-05	0,00860172	-1
**Zfp984**	-2,5559883	0,00335986	0,12062402	-1
**Adamtsl2**	-2,5490947	0,00014311	0,01354503	-1
**Oas1c**	-2,4885447	0,00155037	0,07458153	-1
**Tph2**	-2,4767505	0,00322727	0,11939254	-1

**Gulp1**	-2,4668921	6,322E-05	0,00737192	-1
**Rgs13**	-2,4316281	0,00968495	0,24255646	-1
**Pou2af1**	-2,3769816	0,00320682	0,11893799	-1
**Mtx2**	-2,3266948	0,00065421	0,04111302	-1
**Oasl2**	-2,3191115	9,5394E-07	0,00035653	-1
**Thbs3**	-2,3041706	6,0197E-12	2,6489E-08	-1
**Gabrg1**	-2,284064	0,00047118	0,0320929	-1
**Adcyap1**	-2,283297	0,00047758	0,03237743	-1
**Olfm3**	-2,2775222	0,00530505	0,16348089	-1
**Grb10**	-2,2586334	0,00026964	0,0217146	-1
**Pdlim3**	-2,2420111	1,9476E-08	1,5772E-05	-1
**Zfp931**	-2,2305035	0,00904358	0,23126176	-1
**Oasl1**	-2,217783	0,00153178	0,0739312	-1
**Xaf1**	-2,2146252	0,00436513	0,1442769	-1
**Rimbp2**	-2,2064475	0,00022343	0,01882518	-1
**St8sia4**	-2,194399	0,00498083	0,157144	-1
**Hba-a1**	-2,146692	0,00948189	0,2387013	-1
**Mx2**	-2,1466197	0,00124656	0,06512491	-1
**Gpr34**	-2,1046766	0,00855044	0,22282031	-1
**mt-Nd6**	-2,0733015	0,00584856	0,17540861	-1
**Zfp54**	-2,0709822	0,00107499	0,05835721	-1
**Insm2**	-2,0564026	0,00869635	0,22395403	-1
**Adam18**	-2,0532944	0,00347078	0,12364212	-1
**Gsto1**	-2,0498252	2,2847E-06	0,00069379	-1
**H2-Q6**	-2,0331474	0,00869133	0,22395403	-1
**Rnf213**	-2,0327889	2,7257E-09	3,0562E-06	-1
**Rtp4**	-2,0147668	0,00084559	0,04871656	-1
**P2rx3**	-2,004993	0,00070073	0,0427123	-1
**Pcsk9**	-1,9907234	0,00381673	0,13093394	-1
**Zfp758**	-1,9552816	0,00666184	0,18998929	-1
**Ifih1**	-1,9538318	4,9851E-06	0,00121105	-1
**Col27a1**	-1,9481495	0,00234671	0,09773021	-1
**Pcolce2**	-1,9466558	0,00601364	0,17760901	-1
**Ttpa**	-1,9436209	0,00042193	0,02985441	-1
**Isg15**	-1,938764	0,00250854	0,1021353	-1
**Obscn**	-1,9355284	0,00166791	0,07854947	-1
**Ifi27**	-1,9310096	3,8547E-09	3,7457E-06	-1
**Rsad2**	-1,9233431	8,9273E-05	0,00971073	-1
**Ifit3**	-1,9066444	0,00225885	0,09499685	-1
**Cpne4**	-1,9057267	0,00501836	0,15764561	-1
**Frk**	-1,9007788	0,00359456	0,12564582	-1
**Gbp9**	-1,8859923	2,8462E-08	1,9755E-05	-1
**Alx4**	-1,8682361	0,00734714	0,20398471	-1
**Hsd11b1**	-1,8660089	0,00128407	0,06631607	-1
**Adamts19**	-1,8610155	0,00018403	0,01625748	-1
**Aplnr**	-1,8493893	0,00035786	0,02661312	-1
**Gbp3**	-1,8242461	9,1059E-05	0,00983162	-1
**Islr2**	-1,8234979	0,00018187	0,01625748	-1
**Gbp6**	-1,8108644	0,00920617	0,23326315	-1
**Col2a1**	-1,7858909	0,00569082	0,17209412	-1
**Tsacc**	-1,7776551	0,00987825	0,24424096	-1
**Fbln2**	-1,7492908	6,8565E-06	0,00146972	-1
**Igfbp4**	-1,7432477	3,6984E-06	0,00103669	-1

**Nap1l3**	-1,7326961	0,00128963	0,06631607	-1
**Stom**	-1,728082	0,00173077	0,0798708	-1
**Zfp975**	-1,7229731	8,8623E-05	0,00971073	-1
**Ttc23**	-1,7173156	0,00096593	0,05353392	-1
**Ifi35**	-1,697088	0,00014059	0,01339403	-1
**Dtx3l**	-1,6949881	0,00013336	0,0128842	-1
**Gbp7**	-1,6878065	1,9544E-05	0,00317018	-1
**Igtp**	-1,6808901	0,00265271	0,10587879	-1
**Adamts17**	-1,6729769	0,00197927	0,08715974	-1
**Parp9**	-1,6669402	1,092E-08	9,9479E-06	-1
**Serpinh1**	-1,6618315	0,00080034	0,04647294	-1
**Kyat3**	-1,660791	0,0001187	0,01176983	-1
**Slfn8**	-1,6545377	5,3825E-05	0,00653796	-1
**Fhl4**	-1,6490542	0,00535297	0,16426298	-1
**Bche**	-1,6404814	9,653E-05	0,01027027	-1
**Arhgap5**	-1,6383056	0,00332112	0,11976289	-1
**Pvalb**	-1,6360484	0,00330438	0,11976289	-1
**Gpr68**	-1,6236016	0,00067937	0,04231837	-1
**Kcnt2**	-1,5931112	0,00555903	0,16916162	-1
**Plagl1**	-1,5913955	0,000269	0,0217146	-1
**Cebpa**	-1,5901646	0,00199611	0,08751117	-1
**Arxes2**	-1,5888251	3,1374E-05	0,00443982	-1
**Apln**	-1,5825949	0,00119667	0,06340389	-1
**Pcdh19**	-1,5825941	0,00587104	0,1756001	-1
**H3f3aos**	-1,5824496	0,00267183	0,10591048	-1
**Fbn2**	-1,5740483	0,00380672	0,13093394	-1
**Herc6**	-1,5544476	5,5367E-05	0,00666963	-1
**Vegfc**	-1,5477426	0,00605026	0,17777991	-1
**H2-Q7**	-1,5473364	0,00688421	0,19371465	-1
**Itgbl1**	-1,5406802	0,00441258	0,14485993	-1
**Shisa8**	-1,5356198	0,00132624	0,06759197	-1
**Zfp942**	-1,5349843	0,00575884	0,17379045	-1
**Ifit3b**	-1,529962	0,0062178	0,18053925	-1
**Dio2**	-1,5213739	0,00070621	0,0427123	-1
**Nfatc4**	-1,5161937	0,00011661	0,01172187	-1
**Chchd7**	-1,4797764	0,00515492	0,16020918	-1
**Nptx1**	-1,4739088	5,8451E-05	0,00698352	-1
**Car4**	-1,4656229	0,00054619	0,0360616	-1
**Igfbp2**	-1,428466	1,9574E-05	0,00317018	-1
**Kcnn1**	-1,419669	0,00173371	0,0798708	-1
**Ubxn11**	-1,4026981	0,00485415	0,15448504	-1
**Lrif1**	-1,3959155	0,00505803	0,15855015	-1
**Aldh1a1**	-1,3946438	0,00043405	0,03041673	-1
**Col9a3**	-1,3893381	0,00022548	0,01888812	-1
**Onecut2**	-1,3730947	0,00129211	0,06631607	-1
**Mr1**	-1,3669771	0,0008708	0,04977557	-1
**Zxdb**	-1,3525987	0,00245473	0,10059756	-1
**Hopx**	-1,3504354	0,00099077	0,05470228	-1
**Gpr62**	-1,3371947	0,00324226	0,11964341	-1
**Arxes1**	-1,33466	0,00029231	0,02266354	-1
**Tecta**	-1,3278329	0,00752682	0,2070017	-1
**Rbm20**	-1,3195108	0,00839018	0,22035185	-1
**Tdrp**	-1,3086678	0,00230771	0,09665844	-1

**Ntng2**	-1,28019	0,00304041	0,11596476	-1
**Fmod**	-1,2589799	0,00834258	0,21949713	-1
**Kcnj16**	-1,2523963	0,00988626	0,24424096	-1
**Irgm1**	-1,251035	0,00011648	0,01172187	-1
**AW146154**	-1,2494282	0,00107698	0,05835721	-1
**Trim21**	-1,2376918	0,00347761	0,12364212	-1
**Kctd8**	-1,2321885	0,00148719	0,07249907	-1
**Wdpcp**	-1,2309645	1,9E-05	0,00314707	-1
**Tmem47**	-1,2292314	0,00070357	0,0427123	-1
**Plxdc1**	-1,2252607	0,00262312	0,10504011	-1
**Lcat**	-1,2183206	3,7044E-05	0,00501564	-1
**Lpar4**	-1,211707	0,00223996	0,09463689	-1
**Rasd2**	-1,199522	0,0053176	0,16352171	-1
**Fam84a**	-1,1709041	0,00156172	0,07488027	-1
**Papln**	-1,1609215	0,00758366	0,20724675	-1
**Pter**	-1,1599029	0,00974771	0,24329214	-1
**Chn1**	-1,1550009	0,00092838	0,05224709	-1
**Cpxm2**	-1,1499443	0,00409884	0,13766072	-1
**Wwc2**	-1,147005	0,00318685	0,11880173	-1
**Cybrd1**	-1,1355308	0,00200105	0,08751117	-1
**Vcpkmt**	-1,1304834	0,00428694	0,14233802	-1
**Ddx58**	-1,1228787	0,00383887	0,13093394	-1
**Crim1**	-1,1194386	0,00278929	0,10958696	-1
**Eng**	-1,1085351	0,0072618	0,20238612	-1
**Cyp7b1**	-1,1065475	3,2666E-05	0,00457833	-1
**Sardh**	-1,1063791	0,00226152	0,09499685	-1
**Rras2**	-1,0918975	0,00120057	0,06340389	-1
**Socs2**	-1,0899862	0,0030471	0,11596476	-1
**Acsbg1**	-1,0839302	0,00048879	0,03283216	-1
**Hr**	-1,0691839	0,00105909	0,05781777	-1
**C1qtnf6**	-1,0562634	0,00312726	0,11748197	-1
**Rundc3b**	-1,0561424	0,00029026	0,0226251	-1
**Sox2**	-1,0284553	0,00020128	0,01715721	-1
**Kdr**	-1,0166005	0,00332766	0,11976289	-1
**Tap2**	-1,0070268	0,00929257	0,23474611	-1
**Gpd1**	-1,003799	0,00169976	0,07890339	-1
**Efnb2**	-1,0031111	0,00732186	0,20367075	-1
**Tril**	-1,0021816	0,00135752	0,06823185	-1

Related to Figure 2

List S2. List of mouse genes specifically expressed in each SHH molecular subtype

SHHα *vs.* β, γ and δ	SHHβ *vs.* α, γ and δ	SHHγ *vs.* α, β and δ	SHHδ *vs.* α, β and γ
**Tom1l1**	Parm1	Slc17a7	Mycbpap
**Melk**	Dock10	Cbln3	Gcm1
**Phgdh**	Islr2	Kcna1	Rrh
**Pawr**	Ryr3	Gabra4	Eno4
**Pclaf**	Neurod6	Vsnl1	Gcgr
**Chrdl1**	Ebf2	Phyhip	Tppp3
**Adgrv1**	Grin2b	Wscd2	Cfap54
Dach2	Slc22a15	Fat2	BC048671
**Cabp7**	Lhx8	Gpr158	Pde2a
**Car2**	Fstl4	Atp2b3	Nfatc1
**Hey1**	Cbln1	Gabra6	Atp2a3
**Calca**	Igf2bp3	Gabrd	Ppp1r1b
Otx1	Zfy2	Ptprr	C1ql1
**Lmo3**	Zfy1	Chrna6	C2cd6
**Otx2**	Cntn5	Nrxn3	Cyp3a41b
**H3c8**	Nhlh2	Tnr	Cyp3a11
**Dcdc5**	Ndst4	Slitrk4	Cyp3a41a
**Dapl1**	Dlx5	Tubb4a	Cyp3a44
**Myocd**	Cdh7	Mical2	Cyp3a16
	Kcnj3	Micalcl	Cyp3a59
	Sphkap	Chrnb3	Cyp3a57
	Ank3	Grm1	Slc6a11
	Qrfpr	Lgi1	Sfrp5
		Tenm1	Grik3
		Rasgrf1	Plcxd3
		Camk2b	Hoxa3
		Diras2	Dpp4
		Synpr	Hoxa9
		Gpr83	Pde3a
		Scn2a	Kitl
		Arpp21	Rph3a
		Kcnk1	Hoxa2
		Pclo	Hcar1
		Gabbr2	Slc4a11
		Gabrb2	Cntn3
		Vat1l	Popdc3
		Lrp1b	Cdh23
		Fstl5	Spx
		Mal2	Hoxa7
		Car10	Gabrg1
		Grm4	Cldn1
		Sel1l3	
		Cadps2	
		Hcn1	
		Ryr2	
		Tmem163	
		Gabrb1	
		Cdh18	
		Tll1	

		Gabra1	
		Chl1	
		Kcnj3	
		Dock3	
		Sphkap	
		Ptprz1	
		Ank3	

Related to Fig. 2

List S3. List of the 24 mouse genes specifically expressed in SHHα p53 mutant LC/A MBs

SHHα p53 mutant LC/A *vs.* SHHα p53 wildtype desmo/classic

**Bdkrb1**
**Stc2**
**Steap3**
**Rimbp2**
**Tmem185b**
**Fkbp4**
**Kcnq5**
**Tmem177**
**Rgs16**
**Ano5**
**Gal**
**Pmp2**
**Lrrtm1**
**Ckb**
**Pcdhb16**
**Cdh7**
**Lingo2**
**Skap2**
**Fabp6**
**Lhx8**
**Tbx20**
**H2bc18**
**Cdh12**
**Mal2**

Related to Fig. 2
